# Chromium Cation‐Induced Self‐Reconstruction of a Stable and High Performance Boride‐derived Electrocatalyst for Oxygen Evolution Reaction

**DOI:** 10.1002/smll.202507475

**Published:** 2025-09-24

**Authors:** Charles Otieno Ogolla, Max Kasper, Muhammed Fasil Puthiyaparambath, Nastaran Farahbakhsh, Ranjit Thapa, Maiyalagan Thandavarayan, Manuela S. Killian, Benjamin Butz, Jean Marie Vianney Nsanzimana

**Affiliations:** ^1^ Micro‐ and Nanoanalytics Group University of Siegen Paul‐Bonatz‐Straße 9–11 57076 Siegen Germany; ^2^ Department of Physics & Centre for Computational and Integrative Sciences SRM University AP Amaravati Andhra Pradesh 522240 India; ^3^ Chemistry and Structures of Novel Materials University of Siegen Paul‐Bonatz‐Straße 9–11 57076 Siegen Germany; ^4^ Department of Chemistry SRM Institute of Science and Technology Kattankulathur Tamil Nadu 603203 India; ^5^ Department of Industrial Engineering University of Padova Via Marzolo 9 Padua 35131 Italy

**Keywords:** catalyst self‐reconstruction, electrocatalysis, metal boride, microstructure, oxygen evolution reaction

## Abstract

The rational design of efficient electrocatalysts for the oxygen evolution reaction (OER), holds the key to advancing the overall electrolytic water splitting performance. Here, a scalable one‐pot synthesis of a stable chromium─iron nickel boride (Cr─FeNiB) electrocatalyst is reported for OER in which nickel‐boron sites are micro‐environmentally modified through interactions with iron and chromium. Comprehensive, correlative electrochemical, structural, and chemical analyses reveal the formation of amorphous‐crystalline core‐shell structures that transform into nanosheets upon activation with enhanced water oxidation catalytic activity. The enhanced catalytic performance is attributed to the chromium‐induced chemical self‐reconstruction of the catalyst, which facilitates favorable OER kinetics, increased turnover frequency, and a synergistic effect between metal and boron constituents. Density Functional Theory (DFT) calculation showed that chromium incorporation effectively shifts the *d*‐band centers (*ɛ_d_*) closer to the Fermi level and narrows the metal‐d/boron‐p band center gap (Δ*ɛ_d‐p_*) (Ni_2_B 1.10 eV → FeNiB 0.71 eV → Cr─FeNiB 0.55 eV) ultimately enhancing OER activity. Accordingly, the Δ*ɛ_d‐p_* is established as a key electronic descriptor for predicting and optimizing OER performance. These findings pave the way for a better understanding of metal boride‐derived electrocatalysts and also contribute to the development of efficient, stable, earth‐abundant, non‐noble metal catalysts for water oxidation.

## Graphical abstract

A rational design of a highly efficient and stable oxygen evolution reaction (OER) electrocatalyst is achieved by microenvironmental modification of nickel‐boride active sites using cations, synthesized via a scalable one‐pot method. Comprehensive microscopy and spectroscopic characterization consistent with Density Functional Theory (DFT) simulations reveals chemical reconstruction toward a stable metal boride iron‐triad phase that enhances OER kinetics, producessynergistic effects, and provides insights for the development of advanced non‐noble metal‐based electrocatalysts.

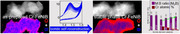

## 1 Introduction

Green hydrogen produced through water electrolysis has become significant in the global efforts to decarbonize energy systems as it represents a carbon‐neutral alternative to fossil fuels. It offers a promising pathway for sustainable and efficient chemical production, low‐carbon fuel in transport, and long‐term energy storage.^[^ ^1^ ^]^ Among the existing technologies, alkaline water electrolysis (AWE) is well established for industrial‐scale hydrogen production with the ability to be operated at comparatively low capital costs.^[^ ^2^ ^]^ Despite these strengths, AWE faces significant challenges that prevent it from economically competing against fossil fuel‐derived hydrogen. Enhancements in durability, efficiency, and cost reduction are essential if electrolytic water splitting technology is to make a meaningful contribution to a sustainable hydrogen economy.

One of the major challenges in the application of conventional AWE is the severe operating conditions. Industrial processes occur under highly concentrated alkaline solutions, typically ≈6 – 7 m potassium hydroxide (KOH), to achieve high ionic conductivity. While these conditions are effective for performance, such concentrated electrolytes are highly corrosive and therefore, make the entire process hazardous, shorten the operational lifetime of the cell components, which ultimately makes maintenance highly demanding and raises the overall costs of operation.^[^ ^3^ ^]^ Newer approaches, such as anion exchange membrane water electrolysis (AEMWE) offer alternatives designed to mitigate these challenges by enabling efficient operation even at much lower alkaline concentrations (≤1 m KOH).^[^ ^4^, ^5^ ^]^ Hence, investigating the OER performance in 1.0 m KOH is ideal. Regardless of the configuration, however, OER at the anode still remains the primary bottleneck affecting the efficiency of electrolytic water splitting.

The OER process is inherently complex as it requires the transfer of four electrons and four protons along with the O─H bond cleavage and O─O bond formation. Specifically in alkaline media, this process in the adsorbate evolution mechanism (AEM) is facilitated by the surface adsorption and transformation of intermediates (OH^*^, O^*^, OOH^*^) at the active metal site on the catalyst surface.^[^ ^4^, ^6^ ^]^ The formation of O─O bonds may proceed by either the coupling of adjacent O^*^ or through an OOH^*^ intermediate evolution.^[^ ^7^ ^]^

The relative binding energies associated with the formation of these intermediates control the rate‐determining step and therefore the required overpotential. Because optimizing the binding energy for one intermediate may lead to the destabilization of another, these steps are often linked by scaling relationships, which result in the sluggish kinetics of OER during water splitting. In order to overcome this intrinsic limitation, catalysts need to carefully balance the binding energies of the intermediates to lower the kinetic barrier. Noble metal oxides such as ruthenium oxide (RuO_2_) and iridium oxide (IrO_2_) based materials remain the benchmark catalysts for OER even in a wide pH range.^[^ ^8^, ^9^ ^]^ However, their high costs and scarcity exclude them from cost‐effective deployment in large‐scale applications. Research focus has therefore shifted toward the development of catalysts based on earth‐abundant elements, with design strategies such as electronic tuning, nano structuring, and compositional doping being applied to achieve enhanced OER activity.^[^ ^10^, ^11^ ^]^

In this respect, nickel‐based materials are among the most promising as economically viable and abundant materials for hydrogen production in alkaline media.^[^ ^4^, ^12^, ^13^ ^]^ Nickelis inexpensive, stable under alkaline conditions, and can form compounds in different valence states (valency tunable). The multiple oxidation states facilitate redox transitions, which are important in OER catalysis.^[^ ^7^ ^]^ This allows for the nickel compound to reconstruct during operation to form oxyhydroxide phases at the surface, which sustain the catalytic activity. Such kind of restructuring is sometimes described as a self‐healing mechanism and is evident in first‐row transition‐metal oxide catalysts.^[^ ^14^ ^]^ Still, the performance of such Ni‐based electrocatalysts is not fully understood and does not fulfill the requirements for an ideal OER electrocatalyst, necessitating further improvements.^[^ ^15^ ^]^

Different strategies are implemented to enhance the performance of these catalysts, including modulation of chemical composition, crystalline structure, morphology, electrical conductivity, and surface chemistry, targeting the changes that occur during the OER operation.^[^ ^15^ ^]^ Chemical modulation involves changing the active‐site (Ni) chemistry by the strategic introduction of metal cations (e.g., the iron triad elements) or non‐metal anions forming nitrides, phosphides, chalcogenides, borides, and borates. Those Ni‐modulated catalytic materials exhibit enhanced electrical conductivity, charge transfer (donating/accepting (e‐DA) ability), and surface ad(de)sorption properties.^[^ ^15^ ^]^ Such catalysts need to be robust, stable, and sustain catalytic activity even after structural reconstruction, which occurs in situ under oxidative conditions to form an active metal‐oxyhydroxide amorphous phase upon which water oxidation occurs (OER).^[^ ^16^, ^17^ ^]^

Non‐metal anion incorporation, especially boron, has proven to be particularly effective at enhancing the activity of Ni‐based catalysts. Boron regulates the electronic structures of metals due to its unique metalloid properties.^[^ ^18^ ^]^ The high electronegativity of boron alters the local electronic environment of Ni, lowering the energy barrier of nickel boride phase active sites (Ni and B) during the water oxidation reaction and facilitating charge transfer.^[^ ^19^, ^20^, ^21^ ^]^ In fact, it has been reported that the presence of guest atoms within the Ni lattice induces strain and adjusts the bond distances, which also enhances the catalytic activity of the electrocatalyst.^[^ ^19^ ^]^ Structure modification strategies have been successfully utilized to enhance the catalytic performance of nickel boride catalysts. The overall (multi)metal to boron ratio is critical in catalyst performance as deviations from the stoichiometric balance have been determined to also play a fundamental role in the ultimate performance and stability of the catalyst, especially in OER.^[^ ^22^ ^]^ For instance, Ni_2_B showed increased catalytic performance upon moderate annealing, while prolonged annealing even became detrimental.^[^ ^23^ ^]^ Through this energy‐consuming process, the catalytic activity of Ni_2_B reaches an optimum value, and further heat treatment leads to the formation of a crystalline phase and the formation of the Ni_3_B phase with decreased catalytic activity.

Another powerful approach to enhance nickel boride‐based catalysts is the incorporation of transition metals with compatible structures due to synergistic effects among these elements.^[^ ^24^ ^]^ In particular, incorporation of ironin nickel boride at a lower ratio (metal:boron ratio of 2:1) has shown improvement in the catalytic activity of nickel boride.^[^ ^20^, ^25^, ^26^ ^]^ Iron is widely used because it provides electronic flexibility, which modulates the activity of adjacent Ni centers. This cooperative interaction between Fe and Ni sites significantly enhances the activity and stability of the Ni catalyst by its dual‐metal‐site nature.^[^ ^4^, ^27^, ^28^ ^]^ While such FeNi‐borides already show strong promise as OER catalysts, opportunities still remain for further improvements by incorporating additional metals that form hard and boron‐rich boride phases (stoichiometric metal‐to‐boron (M:B) ratio 1:1 or 1:2, respectively).^[^ ^29^ ^]^ Chromium is one such promising candidate in which high valence Cr atoms can be doped in the FeNi‐boride structure. It exhibits a large range of valence states (−2 to +6) and is known to form borides with high stability, which leads to a desirable modulation of the FeNi‐boride active sites.^[^ ^30^ ^]^ Chromium incorporation, therefore, has the potential to induce lattice strain, alter local coordination environments, and encourage the beneficial surface reconstruction during operation. Additionally, cation leaching of Cr under OER conditions may result in the creation of oxygen vacancies, which serve to stabilize active oxyhydroxide phases. According to our knowledge, while multi‐metallic M_2_B stable phases have been widely investigated, rational catalyst‐structure design for the successful inclusion of high valency Cr (which deviates from their known M:B ratio 2:1) into the complex FeNi‐boride structure to develop a well‐performing electrocatalyst for OER has not been reported so far.

This work addresses that gap by developing a Cr─FeNiB catalyst synthesized via a scalable, cost‐effective (annealing‐free) reduction process. Through the incorporation of Cr into FeNi‐boride, the local electronic environment and metal‐to‐boron ratio are modulated to achieve a controlled reconstruction into a stable and dual active phase. A combination of correlative structural, chemical composition, and electrochemical analyses was used to demonstrate how Cr incorporation influences composition, morphology, and OER performance. The results show that enhanced OER kinetics, increased turnover frequency, and improved self‐reconstruction are all favored by the incorporation of Cr, especially due to its high dissolution capacity. The optimized Cr─FeNiB electrocatalyst even achieved overpotentials of only 252 and 272 mV on a carbon paper and glassy carbon electrode, respectively at 10 mA cm^−2^ in 1 m KOH even surpassing the benchmark OER catalyst, RuO_2_, under similar conditions. DFT calculations demonstrated that the Cr further modified the electronic boride and the calculated *d*‐*p* band difference (∆*ɛ_d‐p_* = *ɛ_d_ – ɛ_p_*) following the trend Ni_2_B (1.10 eV) < FeNiB (0.71 eV) < Cr─FeNiB (0.55 eV). Beyond the reporting of a promising electrocatalyst, this work provides an experimentally‐grounded picture of the reconstruction phenomena in a boride‐derived system and presents a broader strategy that can be utilized for the design of stable, efficient, and earth‐abundant electrocatalysts for water oxidation.

## 2 Results and Discussion

### 2.1 Catalyst Synthesis and Composition

**Figure 1:**
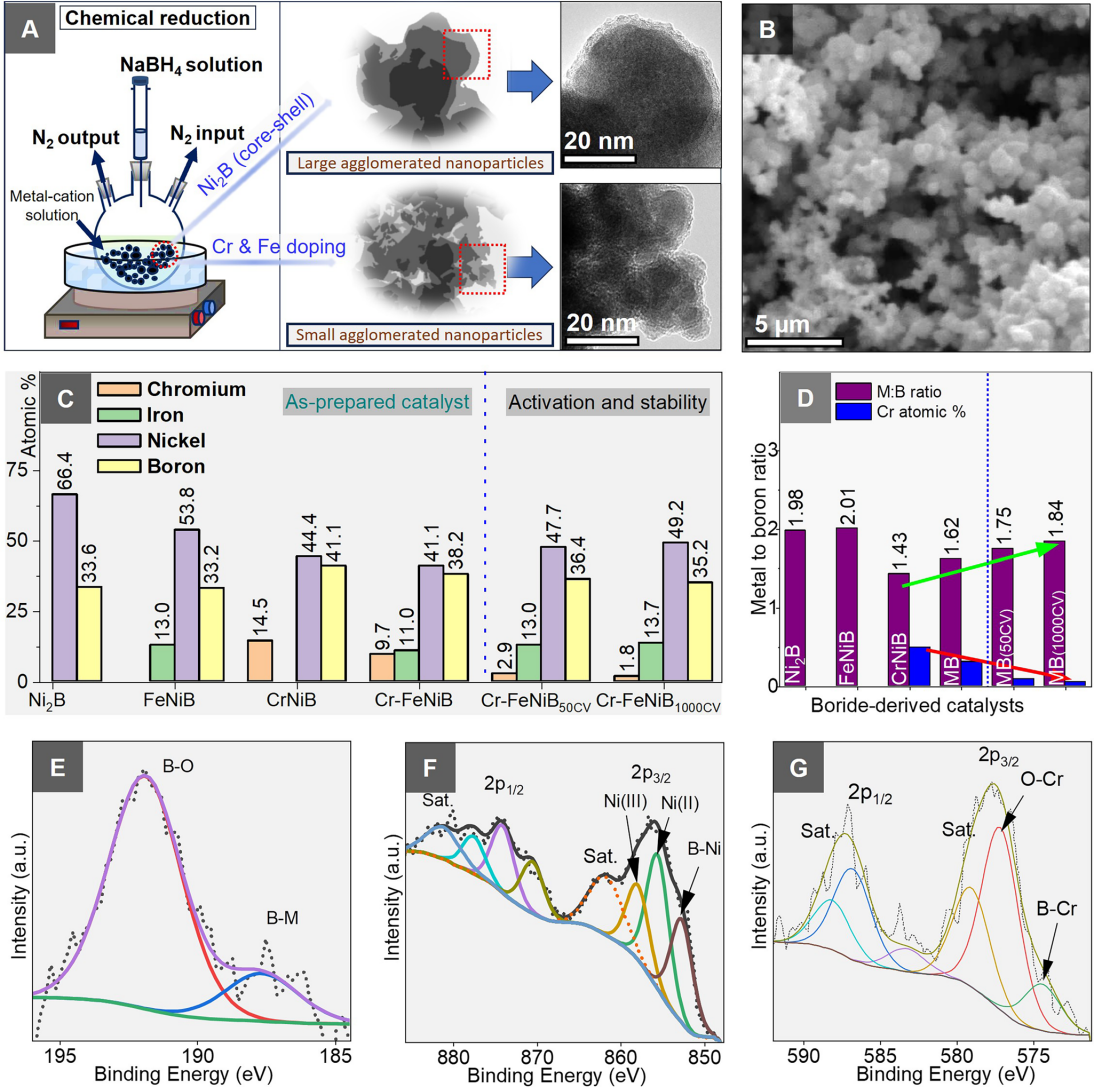
Synthesis and surface analysis: A) Schematic of fabrication of high valence chromium and iron inclusion in Ni_2_B‐derived OER catalyst; B) SEM micrographs of catalyst; C) ICP‐OES analysis of a series of Ni_2_B‐derived materials: Ni_2_B, FeNiB, CrNiB, as‐prepared Cr─FeNiB, Cr─FeNiB, and Cr─FeNiB_1000CV_; D) Analysis of chromium and iron incorporation on the Ni_2_B to form metal boride (M_2_B). MB stands for Cr─FeNiB, green arrow indicates increasing M:B ratio, red arrow indicates decreasing chromium concentration; E–G) High resolution XPS spectra for B 1s, Ni 2p, and Cr 2p of the as‐prepared Cr─FeNiB.

A series of nickel boride‐based catalysts was systematically synthesized using a scalable and one‐pot chemical reduction method in aqueous media (**Figure** **1**A). In this approach, transition metal ions (e.g., Ni and Fe) were reduced by sodium borohydride (NaBH_4_) solution, producing metal borides with a typical metal‐to‐boron ratio (M:B) of 2:1 (M_2_B) according to the equation (1).^[^ ^23^, ^31^ ^]^

(1) $$2{\mathrm{NiCl}}_{2}+4{\mathrm{NaBH}}_{4}+9H_{2}O\;\to \;{\mathrm{Ni}}_{2}B+4\mathrm{NaCl}+12.5H_{2}+3B{\left(\mathrm{OH}\right)}_{3}$$

In this strongly exothermic reaction, the metal precursors are not only reduced but also boron atoms are availed, which are incorporated into the growing Ni─B framework. During the reduction process, NaBH_4_ acts both as a reducing agent and as a boron source, allowing for the direct formation of nickel boride‐derived materials in the presence of excess borohydride.^[^ ^31^ ^]^ It is established that the inclusion of minor quantities of additional transition metals into the nickel boride lattices significantly enhance its catalytic activity. Therefore, this principle was used in the preparation of a series of ternary electrocatalysts (FeNiB, CrNiB, CoNiB, MnNiB, and ZnNiB (χ_M_ = 0.2), see Supporting Information, material synthesis section).

Enhanced catalytic performance was consistently observed for materials containing Fe and Cr compared with the other ternary materials within the tested series (Figure S6, Supporting Information). These findings were used as the basis for the design of trimetallic catalyst where both Fe and Cr are included into Ni_2_B to yield the OER‐optimized electrocatalytically active quaternary Cr─FeNiB system. The resulting catalysts are referred to “as‐prepared Cr─FeNiB ” after the synthesis, Cr─FeNiB after activation (50 CV scans), and Cr─FeNiB_1000CV_ after 1000 CV scans. In order to further demonstrate the dual‐functionality of boron, not only as a reducing agent but also as a metalloid incorporated into the catalyst structure to enhance activity, we prepare complementary multi‐metal oxide and alloy with the same overall metal composition as the optimized catalyst.

Scanning electron microscopy (SEM), revealed extensive nanoparticle agglomeration, consistent with the spontaneous and strongly exothermic nature of this reaction (Figure 1B). To evaluate the effectiveness of this method in preparing metal borides, composition and elemental ratios were determined by inductively coupled plasma optical emission spectroscopy (ICP‐OES) (Figure 1C). Specifically, emphasis was placed on the M:B ratio and relating this to the material's catalytic activity as both metals and B are involved in the OER. The nickel boride sample exhibited an atomic composition of 66% (Ni) and 34% (B), corresponding to an M:B ratio of 1.98, confirming the stoichiometric formation of nickel boride (Ni_2_B). Incorporating Fe in Ni_2_B resulted in a comparable M:B ratio of 2.0 with atomic fractions of 54% (Ni), 13% (Fe), and 33% (B), which is also consistent with the M_2_B structure. These ICP‐OES results are in agreement with the used notation based on the sum of the molar ratios of the precursor metal salts (e.g., Fe_0.4_Ni_1.6_B, hereafter FeNiB).

In contrast, inclusion of chromium produced the lowest M:B ratio, 1.43 for the Cr_0.4_Ni_1.6_B sample. Based on the anticipated 2:1 (metal‐to‐boron ratio) in Ni_2_B, the calculated boron fraction of 22% was exceeded based on 44% of nickel in Cr_0.4_Ni_1.6_B (Figure 1C). The excess boron (19%) is ascribed to chromium (15%), indicating the formation of a chromium boride (CrB_x_). We observe a 1:1.3 metal‐to‐boron ratio indicating CrB_1.3_ formation, which is a clear deviation from the anticipated 2:1 ratio as already observed in Ni_2_B and Fe_0.4_Ni_1.6_B structures. This observation is consistent with the well‐known reduction reactions of chromium salts with borohydride to form CrB_x(1 ≤ x ≤ 2)._^[^ ^27^, ^32^ ^]^ The direct influence of incorporating chromium atoms in the as‐prepared Cr─FeNiB catalyst structure is first proven by comparing the nickel and boron composition in the ternary FeNiB and CrNiB model systems.

Both FeNiB and CrNiB contain comparable amounts of iron or chromium (≈13 – 14.5%) but the nickel fraction decreases significantly from 54% in FeNiB to 44% while B fraction increases when chromium is introduced to form CrNiB (Figure 1C), due to the inclusion of B‐rich Cr‐boride. The Cr─FeNiB quaternary phase also showed a further reduced metal‐to‐boron ratio of 1.62. It is equally important to note that even in this as‐prepared complex quaternary boride system (Cr─FeNiB) a similar divergent M:B ratio is observed with a local Cr:B ratio consistent with CrB_1.25_ (Figure 1D). This deviation from the M_2_B framework is attributable to the incorporation of guest atoms (chromium) whose atomic radius and valence tunability differ from those of other iron triad elements (i.e., nickel and iron).^[^ ^33^ ^]^

Electrochemical activation induced significant structural and chemical transformation of the as‐prepared Cr─FeNiB catalyst. After 50 CV cycles, chromium fraction drastically reduced from 9.7% in the as‐prepared catalyst to 2.9% in the activated catalyst (Cr─FeNiB), boron fraction slightly decreased from 38.2% to 36.4% while nickel and iron fractions remain relatively unchanged. The M:B ratio was noted to increase from 1.62 (as‐prepared Cr─FeNiB) to 1.75 (activated Cr─FeNiB), which is in agreement with the reconstruction (due to the suited atomic radius and electronic structure, as well as the synergistic interplay between the iron triad elements, Ni and Fe) to form the stable M_2_B phase catalyst. The reduction in boron fraction is not entirely explained by the presence of CrB_1.25_ but also highlights the presence of extra boron species in the metal oxyhydroxide surface of the activated phase, which act as the active sites for OER.^[^ ^25^, ^34^ ^]^ After 1000 CV oxidation‐reduction cycles (Cr─FeNiB_1000CV_), the catalyst stabilized with a chromium fraction of ≈2% while Fe and Ni fractions remained relatively unchanged, resulting in an M:B of 1.84, close to the stable M_2_B phase of FeNi‐ and Ni‐boride. This change between the stable phase (Cr─FeNiB_1000CV_) is comparable to that of the activated material (Cr─FeNiB) and not as drastic as that seen between the as‐prepared catalyst and activated material. Notably, however, residual chromium persists even after extended cycling, which shows it still plays a significant role in the catalytic performance of the Cr─FeNiB ‐derived catalyst.

Surface sensitive X‐ray photoelectron spectroscopy (XPS) directly confirmed the presence of Fe, Cr, Ni, O, and B in the XPS‐survey spectrum (Figure S1A, Supporting Information). Formation of boron‐metal bonds and oxidized surface boron species (B^0^/ B – M) is evidenced from the B 1s spectrum, which has two distinct peaks at 187.6 and 191.9 eV, respectively (Figure 1E).^[^ ^18^ ^]^ The Ni 2p spectrum revealed characteristic metal‐boron interactions inferred from the sub peaks in the 2p_2/3_ peak (Figure 1F). The sub‐peak at 852.5 eV is attributed to boride formation, while the sub‐peaks at 855.5 and 857.9 eV correspond to surface Ni^2+^/Ni^3+^ oxide and hydroxide species.^[^ ^23^ ^]^ To check the chemical position of the Ni 2p, a reference spectrum was recorded from a sputter‐cleaned Ni foil (Figure S1D, Supporting Information). The spectrum showed the expected Ni 2p_3/2_ and 2p_1/2_ components at 853.2 and 870.1 eV, respectively, with satellite peaks at 858.7 and 873.7 eV. Deconvolution reveals two doublet peaks, which correspond to minor Ni^2+^ contribution at 855.3 and 871.4 eV. Relative to the as‐prepared material, the Ni 2p_3/2_ component is located at 0.7 eV higher, consistent with the modulation of the Ni electronic structure in the boride environment.

Deconvolution of the Fe 2p spectrum (Figure S1B, Supporting Information) indicated the presence of metal‐boron interaction with a peak at 705.9 eV, while a peak at 711.1 eV confirmed the presence of oxidized Fe species.^[^ ^35^, ^36^ ^]^ Similarly, the Cr 2p spectrum (Figure 1G) exhibited a boron‐metal interaction by a sub‐peak at 744.4 eV and an oxidized chromium species by a peak at 578.5 eV after the 2p_3/2_ spectrum was deconvoluted.^[^ ^37^ ^]^ The O 1s spectrum was deconvoluted into three components (Figure S1C, Supporting Information). The oxygen‐metal (O─M) bond was identified as the source of the peak at the lower binding energy of 528.7 eV, whereas the metal‐hydroxide (M─OH), B─O, and surface adsorbed water correspond to the peaks at 531.3 and 533.1 eV.^[^ ^23^, ^38^ ^]^

### 2.2 Evolution of Morphology, Microstructure, and Chemistry of Catalyst

**Figure 2:**
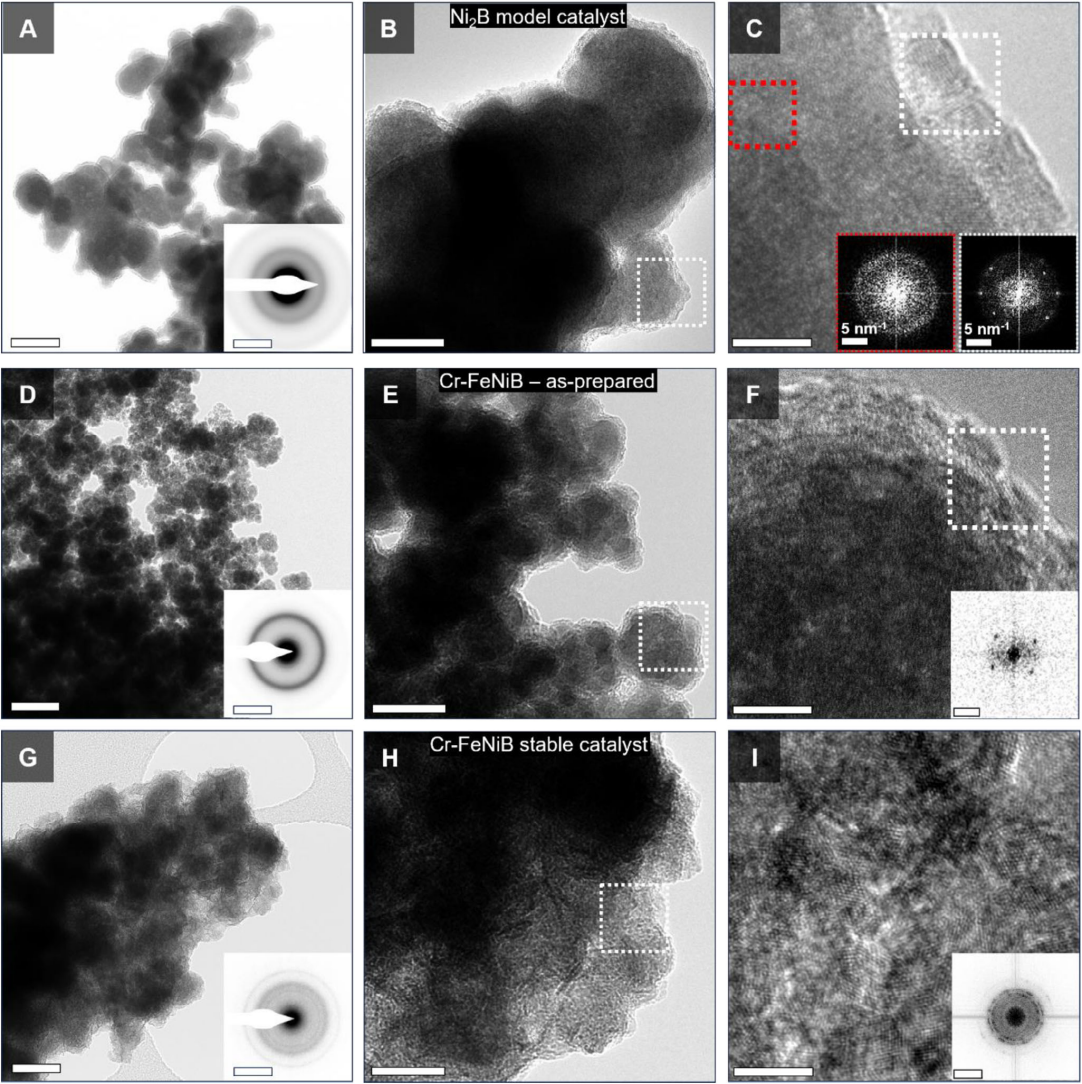
Structural and morphological evolution: A, D, and G) Bright‐field TEM (BFTEM) micrographs showing morphological differences between Ni_2_B (model catalyst), as‐prepared Cr─FeNiB, and stabilized Cr─FeNiB_1000CV_ catalyst (scale bar 50 nm). Insets show SAED patterns indicating structural order (scale bar 5nm^−1^); B, E and H) BFTEM micrographs of core‐shell structures in Ni_2_B catalyst (B) and as‐prepared Cr─FeNiB (E) as well as nanosheets morphology in stabilized Cr─FeNiB_1000CV_ catalyst (H) (scale bar 20 nm); C, F and I) High resolution TEM (HRTEM) micrographs of respective regions marked in B, E and H demonstrating amorphous/nanocrystalline domains in Ni_2_B and as‐prepared Cr─FeNiB, and crystalline ordering in Cr─FeNiB_1000CV_ (scale bar 5 nm). Insets show power spectra of selected regions in C, F, and I (scale bar 5 nm^−1^).

The microstructural and chemical transformations of the catalyst during synthesis and activation play a significant role in the ultimate performance of the catalyst. In the case of the Cr─FeNiB system, chemistry and structural changes were systematically examined in detail by bright‐field transmission electron microscopy (BFTEM), high‐resolution TEM (HRTEM), and selected‐area electron diffraction (SAED) (**Figure** **2**A–I). Together, these analyses establish a clear link between the crystal structure, morphology, and the observed electrocatalytic performance of the catalyst.

Both the Ni_2_B model catalyst and as‐prepared Cr─FeNiB exhibit extensive and rough, nanostructured particulate morphologies (Figure 2A,D) as a consequence of the highly localized exothermic reduction reaction of the respective salts by NaBH_4,_ favoring rapid nucleation and irregular growth. After extended cycling, the active catalyst evolved into extended nanosheet‐like structures on the surface (Figure 2G). BFTEM reveals core‐shell architectures in Ni_2_B and the as‐prepared Cr─FeNiB catalyst (Figure 2B,E), whereas the stabilized, Cr─FeNiB_1000CV_ adopts a more uniform nanoscale sheet‐like morphology without such variations (Figure 2H). HRTEM analysis of representative regions confirms the presence of an amorphous inner core with embedded nanocrystalline domains in both the model Ni_2_B catalyst and the as‐prepared Cr─FeNiB samples (Figure 2C,F and insets), which are hardly reflected in the X‐ray diffraction data (Figure S2, Supporting Information). In contrast, the stable‐phase, Cr─FeNiB_1000CV_ catalyst nanosheets are largely crystalline (Figure 2I, Figure S11, Supporting Information). This structural evolution plays a crucial role in the functionality of the catalyst: the amorphous core ensures high defect site density that can act as active centers for enhanced catalytic activity, while the crystalline domain components impart local order responsible for enhanced electronic conductivity. These transition in structure is confirmed by SAED patterns of representative regions of each of the samples. Ni_2_B and the as‐prepared Cr─FeNiB show diffuse, broad rings characteristic of disordered structures (Figure 2A,D insets).

The diffraction rings of both Ni_2_B and the as‐prepared Cr─FeNiB catalyst correspond to real space distances of ≈0.20 nm, which resembles the interatomic Ni‐B bond distance.^[^ ^39^ ^]^ In contrast, however, the stabilized Cr─FeNiB_1000CV_ catalyst displays more distinct and sharper rings (Figure 2G inset) in addition to the broad Ni_2_B‐like ring, reflecting the emergence of long‐range order and increased crystallinity. These rings correspond to real space distances of 0.25 and 0.15 nm and could be attributed to phases associated with compounds of B‐included metal (Fe, Ni, and Cr) species such as oxides or hydroxides.

The coexistence of amorphous regions and nanocrystalline domains is advantageous since the amorphous matrix provides a high density of coordinatively unsaturated catalytically active sites, while the nanocrystalline domains enhance charge transport/conductivity and structural stability. Synergistically, these features are key for superior OER catalytic activity, as they balance rapid electron transport and structural integrity under the harsh alkaline oxidative conditions.

**Figure 3:**
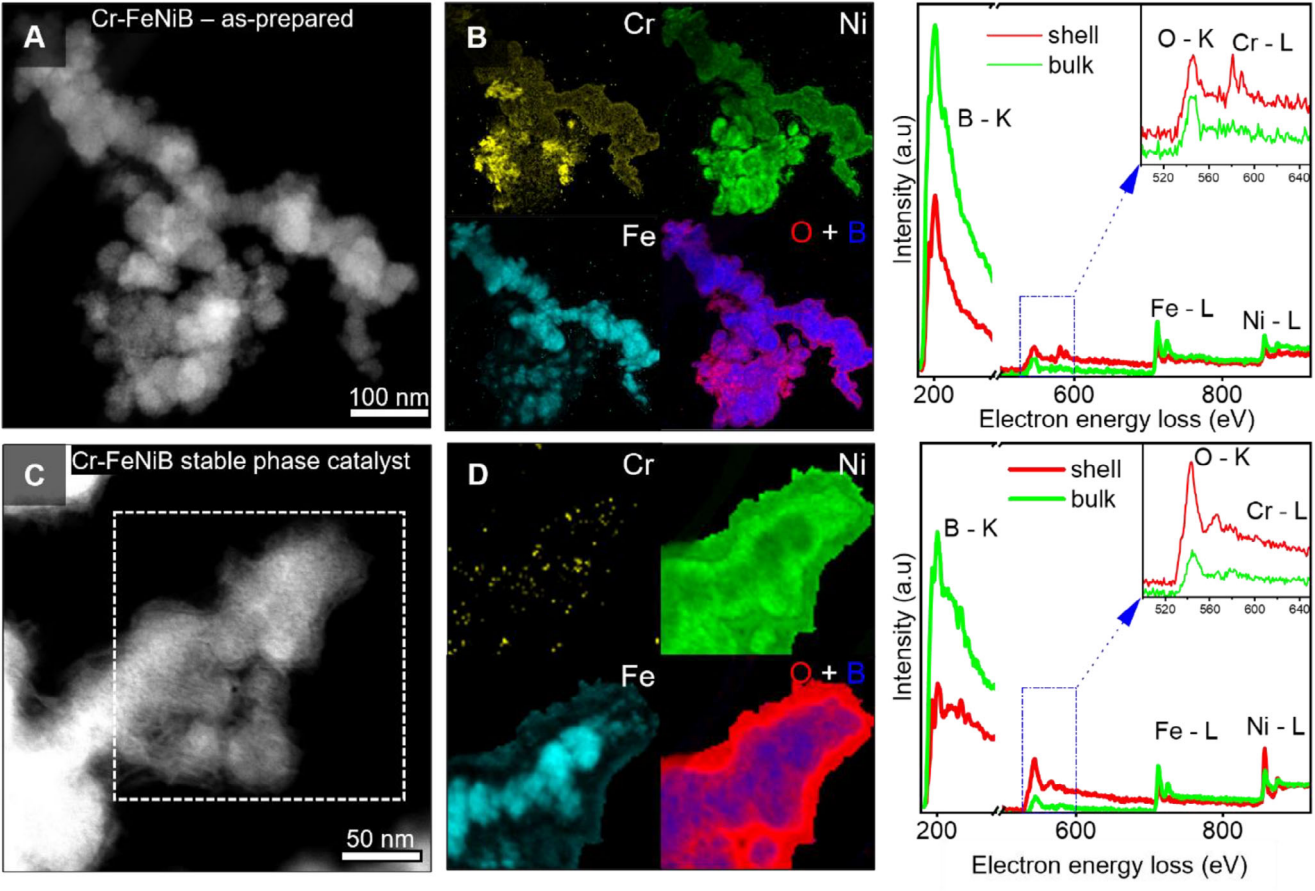
Elemental distribution and chemical evolution of catalysts analyzed by STEM–EELS: A,C) HAADF‐STEM micrographs of selected regions from as‐prepared Cr─FeNiB (A), and stabilized Cr─FeNiB_1000CV_ (C) catalysts; B,D) corresponding EELS elemental maps for Cr, Ni, Fe, and composite O & B distributions with representative EEL spectra shown on the right.

The local spatial distribution of the constituent elements in the as‐prepared Cr─FeNiB (HAADF‐STEM in **Figure** **3**A) and the final activated, stabilized, Cr─FeNiB_1000CV_ (HAADF‐STEM in Figure 3C) was mapped by scanning transmission electron microscopy coupled with electron energy loss spectroscopy (STEM – EELS).

In both materials, the core regions are enriched in B, Ni, and Fe, while the shell shows higher oxygen concentrations (Figure 3B,D), consistent with surface oxidation. In the as‐prepared catalyst, however,  Cr is markedly depleted in the core while the shell exhibits higher Cr concentrations, revealing a local gradient (see spectra and maps, Figure 3B,D, and Figure S3, Supporting Information). Upon activation, the stabilized phase (Cr─FeNiB_1000CV_) catalyst shows substantial Cr leaching, down to below the EELS detection limit in both the bulk and shell regions, while oxygen concentration remains higher at the catalyst surface (Figure 3D and Figure S4, Supporting Information). These chemical gradients highlight the adaptability and chemical versatility of the system and its evolution toward the formation of phases that host OER‐relevant active sites. Cation leaching, e.g. of Cr, and the associated structural reconstruction lead to the creation of oxygen vacancies and induce lattice strain; such relaxation effects have been linked to the preferential formation of 2D nanosheets (Figures 2G and 3C).^[^ ^10^ ^]^

Considered together, BFTEM and SAED define the materials' morphological and structural changes from synthesis across activation and stabilization, while HRTEM and the accompanying FFTs resolve the nanoscale ordering and reconstruction of the catalyst. EELS further co‐locates the corresponding chemical changes with a high spatial precision and proves a higher density of surface B‐metal – oxygen sites that is favorable for improved OER.

### 2.3 Electrocatalytic Performance

**Figure 4:**
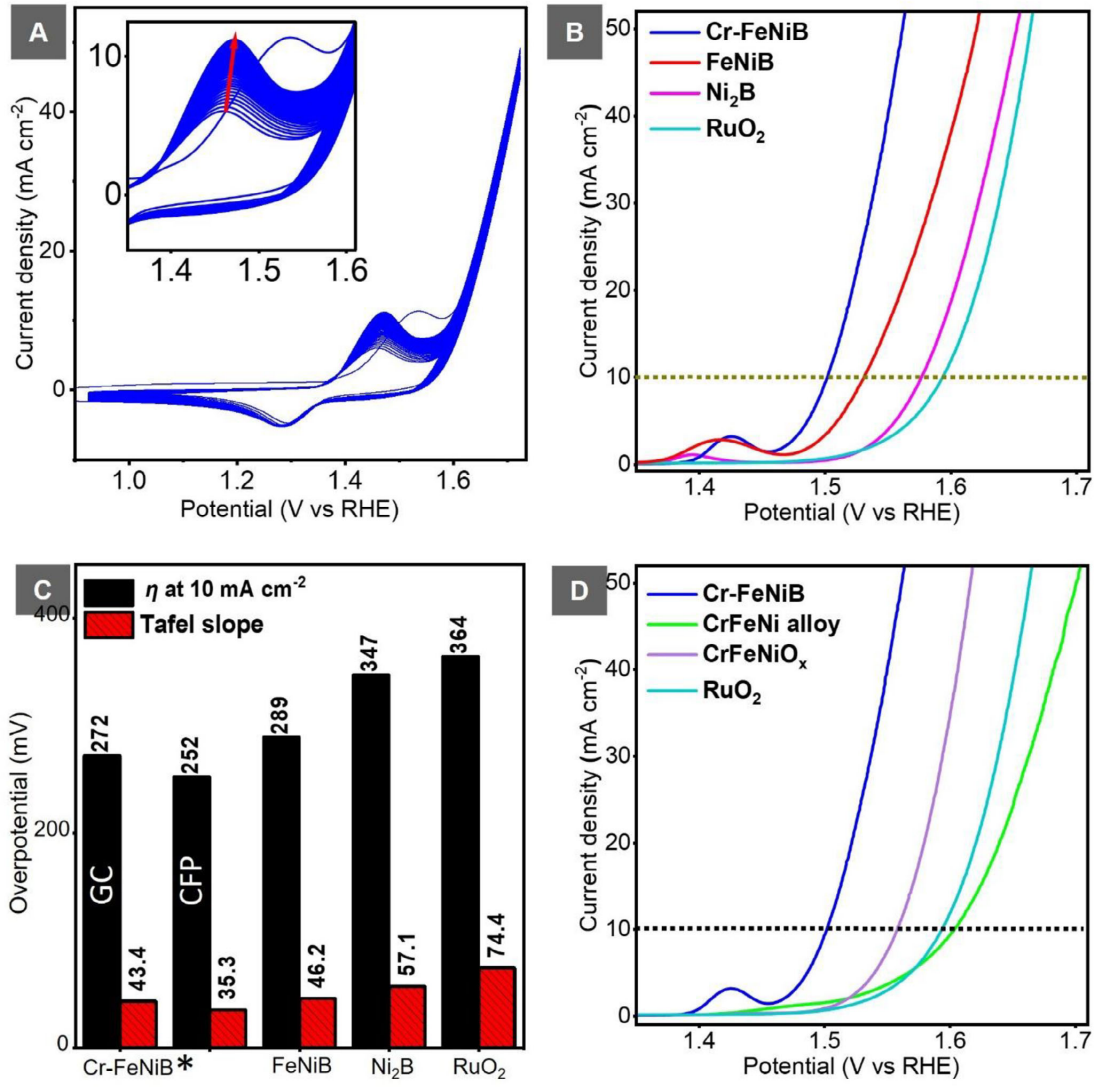
Electrocatalytic activity of Ni_2_B‐derived catalysts compared to RuO_2_: A) activation of as‐prepared Cr─FeNiB by cyclic voltammetry (CV) to active Cr─FeNiB, curves shown without iR correction; B) iR‐corrected LSV polarization curves of Ni_2_B, FeNiB, Cr─FeNiB, and RuO_2_; C) comparison of overpotentials at 10 mA cm^−2^ and corresponding Tafel slopes. ^*^Performance of Cr─FeNiB is herein reported on glassy carbon (GC) and carbon paper (CP), respectively; D) OER performance comparison of optimized Cr─FeNiB with CrFeNi alloy and CrFeNiO_x_ analogues, highlighting the role of boron incorporation to enhance OER activity.

The electrocatalytic performance of the Ni_2_B‐derived catalysts for water oxidation was evaluated in 1.0 m KOH using a standard three‐electrode configuration. A mercury/mercury oxide (Hg/HgO) reference electrode and carbon rod counter electrode were employed, while either a glassy carbon rotating disc electrode (GC/RDE, with a geometric area of 0.196 cm^2^) or carbon paper (CP) loaded with the catalysts served as the working electrode. The series of nickel boride‐derived catalysts used in this investigation required 50 cyclic voltammetry (CV) cycles for activation to obtain reproducible CV curves. The anodic redox process is associated with the reversible transition of M^2+^ to M^3+^.^[^ ^23^, ^28^ ^]^ This activation process was necessary for the transformation of the as‐prepared catalyst material to the OER active and stable phase catalyst (**Figure** **4**A, inset). This activation process accounts for the formation of the complex oxidized surface boro‐metal oxyhydroxides, as confirmed by ICP‐OES and TEM analyses. These support the reconstruction of the catalyst surface into a catalytically active phase.^[^ ^25^, ^34^ ^]^

To assess the influence of Fe and Cr, the OER catalytic activity of Ni_2_B, FeNiB, and Cr─FeNiB was compared against that of the commercial RuO_2_ catalyst under identical loading conditions. Comparative linear sweep voltammograms of the catalysts (Figure 4B), showed overpotentials of 347, 281, 272, and 364 mV at the standard current density of 10 mA cm^−2^ (η_10_), for Ni_2_B, FeNiB, Cr─FeNiB, and the reference RuO_2_ on GC/RDE, respectively. These results indicate that incorporation of boron in enhances the OER catalytic activity of nickel‐based catalyst materials relative to the OER gold standard RuO_2_ catalyst. Further improvements are associated with the incorporation of Fe and Cr as evidenced by the decrease in overpotential upon their introduction into the system (Figure 4B and Figures S5–S7, Supporting Information). Similarly, improved kinetics are observed in the progressive decrease in the Tafel slope values: RuO_2_ (74.4 mV dec^−1^) > Ni_2_B (57.1 mV dec^−1^) > FeNiB (46.2 mV dec^−1^) > Cr─FeNiB (43.4 mV dec^−1^) (Figure 4C). The enhanced kinetics and reduced overpotential of the Cr─FeNiB catalyst evidence an advanced reaction rate that facilitates the anodic OER (Figure 4C & Figure S5C, Supporting Information). Electrochemical impedance spectroscopy (EIS) confirmed the superior charge transfer properties and revealed the catalytic efficiency at the interface of the developed Cr─FeNiB catalyst. Direct observation of the EIS plot semi‐circle curvatures from Figure S5D (Supporting Information) directly reveals that Cr─FeNiB has the smallest curvature compared to Ni_2_B and FeNiB catalysts. Cr─FeNiB displayed a markedly reduced charge transfer resistance (*R*_ct_) value of ≈3.7 Ω, compared to Ni_2_B (18.6 Ω) and FeNiB (6.5 Ω) (Figure S5D, Supporting Information). Together, these observations demonstrate that Fe and Cr act synergistically with Ni and B to improve the OER kinetics and reduce energy losses, and therefore, the significant improvement in the performance of Ni_2_B‐derived catalysts, especially for OER.

The role of boron incorporation in enhancing the catalytic activity was further clarified by comparing the overpotential of Cr─FeNiB with the alloy (CrFeNi) and oxide (CrFeNiO_x_) counterparts. As depicted in Figure 4D, the boride catalyst required the lowest overpotential, further confirming the beneficial impact of B in tuning the electronic environment of Ni centers, while both synergistically being involved in the active phase of the catalyst. The influence of boron was also still effective even after storage of the catalyst under ambient conditions; the overpotential at 10 mA cm^−2^ on CP increased only marginally, from 252 to 253 mV (Figure S9, Supporting Information). Storage of the catalyst under these conditions favors further oxidation of the catalyst, showing that the inclusion of oxygen in this catalyst does not necessarily negatively impact the performance toward OER.

**Figure 5:**
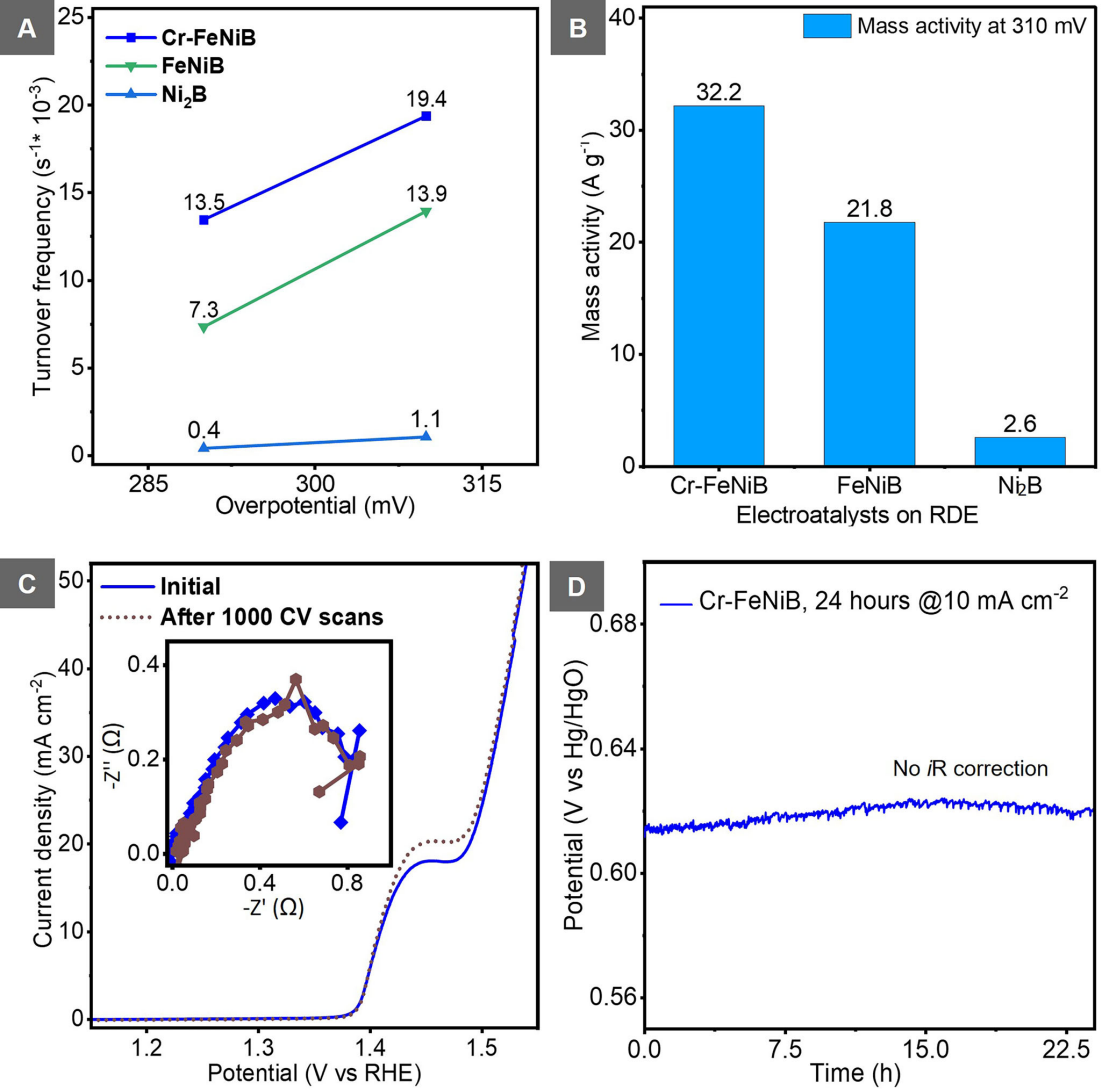
Intrinsic activity and stability of Cr─FeNiB compared to Ni_2_B and FeNiB: A) OER turnover frequency (TOF) values at 310 mV on CFP showing improved intrinsic activity of Cr─FeNiB; B) OER mass activity at 310 mV for the three catalysts; C) stability test of Cr─FeNiB after 1000 CV cycles compared with initial performance. Inset: Nyquist plots from EIS before and after 1000 CV cycles; D) chronopotentiometry of Cr─FeNiB demonstrating long‐term durability.

The incorporation of electron‐deficient B serves as an electron sink, which promotes the Ni^2+^/Ni^3+δ^ redox transition during water oxidation and leading to the formation of boron‐metal oxidized species.^[^ ^21^, ^40^ ^]^ As a consequence, this electronic modulation and its presence in the active phase contribute to the lower overpotentials and improved kinetics during water oxidation compared to the alloy and oxide analogues (Figure S8, Supporting Information). The intrinsic catalytic activity was observed by a marked increase in the turnover frequency (TOF). At 310 mV, Cr─FeNiB exhibited a TOF of 19.4 × 10^−3^ s^−1^ (**Figure** **5**A), which was significantly higher than that of both Ni_2_B (1.1 × 10^−3^ s^−1^) and FeNiB (13.9 × 10^−3^ s^−1^) an indicator that more oxygen was evolved per active site of the optimized catalyst. This improvement highlights the positive effect of multi‐transition metal elements synergy,^[^ ^41^ ^]^ with a total metal ratio of 1.0:4.5:16.4 (Cr:Fe:Ni) in the optimized catalyst material, which enhanced the OER activity even with the drastically reduced chromium content in the catalyst after activation (Figure 1C). Mass activity measurements further confirmed the promising performance of Cr─FeNiB, which achieved 32.2 A g^−1^ in comparison to 21.8 A g^−1^ for FeNiB and 2.6 A g^−1^ for Ni_2_B at 310 mV (Figure 5B).

Durability of the activated Cr─FeNiB was evaluated through accelerated cycling and chronopotentiometry. Even after 1000 CV cycles, Cr─FeNiB still maintained its superior catalytic performance and also showed excellent stability (Figure 5C and inset). This was also proven by the consistency of the chronopotentiometric measurements (Figure 5D and Figure S9, Supporting Information). After accelerated degradation testing (Figure S9, Supporting Information), the overpotential required to achieve a current density of 10 mA cm^−2^ decreased slightly from 252 to 247 mV, indicating enhanced catalyst stability. The difference in overpotentials as measured on GC/RDE versus CP in Figure 4c arises from the variations in mass loading, bubble dynamics, and the corrosion of the substrate, which collectively affect the activity and stability of the catalyst.^[^ ^42^, ^43^ ^]^ GC/RDE measurements are specifically designed to elucidate intrinsic kinetics through the use of thin films, efficient bubble removal, and high mass transport. CP, on the other hand, uses thicker catalyst layers without rotation that more closely simulate realistic water electrolyzer operating conditions. Accordingly, stability testing in this work was conducted on the CP‐coated electrocatalyst, as it more accurately represents catalyst behavior in practical membrane electrode assembly environments.^[^ ^42^, ^44^ ^]^

The higher electrochemical active surface area (ECSA) of the reconstructed catalyst was demonstrated by an increase in the double‐layer capacitance. The activated stabilized Cr─FeNiB catalyst had a larger C_dl_ value of 233 µFcm^−2^ than the as‐prepared Cr─FeNiB catalyst with a value of 133 µFcm^−2^ (Figure S10, Supporting Information). These results confirm that Cr─FeNiB combines high activity, improved kinetics, and long‐term durability, thereby outperforming even RuO_2_ under the same alkaline operating conditions.

### 2.4 Theoretical Calculations

**Figure 6:**
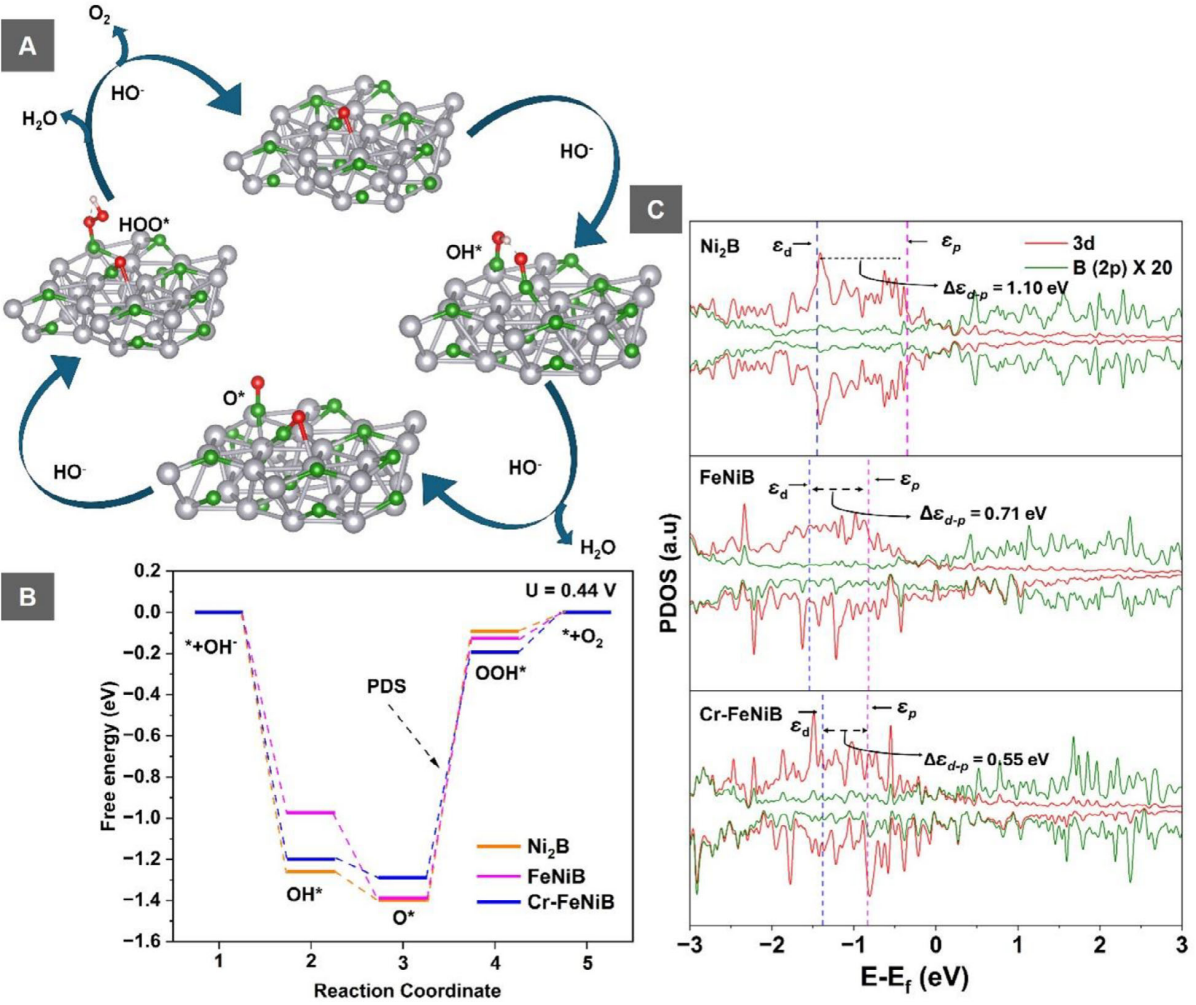
DFT calculations: A) Schematic representation of the OER mechanism on Ni_2_B in the alkaline medium, where Ni (grey), (green), O (red), and H (white); B) Free energy of OER on Ni_2_2B (orange line), FeNiB (magenta line), and Cr─FeNiB (blue line); C) PDOS of *p* and *d*‐orbitals of Boron and transition metals.

To calculate the role of iron and chromium inclusion, Density Functional Theory (DFT) calculations were employed by cleaving the Ni_2_B (001) surface to investigate the adsorption of OER intermediates. The optimized configurations of Ni_2_B, FeNiB, and Cr─FeNiB are shown in Figure S12,Supporting Information. The reaction steps and optimized configurations of the OH^*^, O^*^, and OOH^*^ intermediates on the Ni_2_B surface are shown in **Figure** **6**A. The adsorption of these intermediates was also examined for FeNiB and Cr─FeNiB, with all adsorption steps preferentially occurring at the B sites, synergistically with metal sites. The corresponding free energy profiles for the three systems are shown in Figure 6B. The largest reaction energy barrier, identified as the potential determining step (PDS), corresponds to the third step, which is the OOH formation.

To investigate the role of Cr incorporation in enhancing activity, the projected density of states (PDOS) of Ni_2_B, FeNiB, and Cr‐FeNiB were analyzed and are shown in Figure S12, Supporting Information. The introduction of transition metals induces charge redistribution, with electron transfer from the metal atoms to boron, which is further confirmed by the downward shift of the *p*‐band center (*ɛ_p_*) from the Fermi level, as shown in Figure 6C. Such electronic reconfiguration induced by the transition metal incorporation plays a crucial role in modulating the adsorption behavior of OER intermediates. The *d*‐band centers (*ɛ_d_*) of the transition metals in each system were calculated (Figure 6C), revealing a downward shift of the *d*‐*p* band difference (∆*ɛ_d‐p_* = *ɛ_d_ – ɛ_p_*), following the trend Ni_2_B (1.10 eV) < FeNiB (0.71 eV) < Cr─FeNiB (0.55 eV). Since OER is a charge transfer process, a smaller ∆*ɛ_d‐p_* enhances the on‐plane charge sharing and leads to weaker binding between the catalyst and the oxygen intermediate (which defines the OER efficiency). This reduces the energy barrier of the PDS, thereby improving OER activity.^[^ ^45^, ^46^ ^]^ These results further demonstrate that Cr incorporation effectively tunes the ∆*ɛ_d‐p_*, by shifting *ɛ_d_* closer to the Fermi level and reducing ∆*ɛ_d‐p_*, ultimately leading to enhanced OER activity. Moreover, ∆*ɛ_d‐p_* can be established as a key electronic descriptor for predicting and optimizing OER performance. Overall, these theoretical insights are consistent with experimental observations and show excellent agreement with previously reported findings.^[^ ^47^, ^48^ ^]^

## 3 Conclusion

In summary, we developed a scalable and high performing quaternary amorphous‐crystalline electrocatalyst (Cr─FeNiB) for the OER using a simple annealing‐free chemical reduction route. Correlative chemical, electrochemical, microscopic, and spectroscopic analyses demonstrated that the performance improvements originate from the synergistic interaction of local chemical, structural, and morphological reconstruction processes occurring in‐operando during catalyst activation.

The structural transformation is linked to the spontaneous local exothermic reactions, which generated mixed amorphous‐crystalline domains, while the chemical reconstruction is due to the active leaching of chromium cations, which promotes the formation of surface oxidized boron species and metal oxyhydroxides as active sites. The activated Cr─FeNiB catalyst achieved a significantly low overpotential of only 272 mV at 10 mA cm^−2^ in 1 m KOH, surpassing the benchmark commercial RuO_2_ catalyst under the same conditions. The *d*‐*p* band difference (∆*ɛ_d‐p_* = *ɛ_d_ – ɛ_p_*) exhibits a downward shift from 1.10 eV in Ni_2_B, to 0.71 eV due to the inclusion of Fe, and further by Cr‐inclusion to 0.55 eV in the optimized Cr‐FeNiB system. Since OER involves charge transfer, a smaller ∆*ɛ_d‐p_* enhances on‐plane charge sharing, weakens catalyst–oxygen intermediate binding, lowers the PDS energy barrier, and improves OER activity.

The findings in this work provide valuable insights and guidance to the rational design of stable, high‐performance OER electrocatalysts based on earth‐abundant elements. Beyond reporting a promising material, this work further advances the fundamental understanding of the reconstruction phenomena in boride‐derived systems and contributes to broader strategies for the development of efficient heterogeneous catalysts for water oxidation.

## Conflict of Interest

The authors declare no conflict of interest.

## Author Contributions

C.O.O. and J.M.V.N. contributed equally to this work. J.M.V.N. conceived the concept of the project, designed the experiments, administration, performed data analysis and interpretation, performed electrochemical measurements, supervised, and wrote a manuscript draft, edited, and revised the final manuscript. C.O.O. experimental design, TEM investigation, sample preparation, data analysis and interpretation, manuscript writing, editing, and revision. J.M.V.N., N.F., and M.K. performed ICP‐OES, XPS & XRD analyses. J.M.V.N. and M.K. synthesized materials and performed electrochemical evaluations. M.F.P., R.T., and T.M. performed the DFT calculation, analyzed, and revised the manuscript. B.B. supervised the project, funding acquisition, project administration, data interpretation, manuscript writing, editing, and revision, while M.S.K. sourced project funding and revised the manuscript. All authors contributed to the discussion and editing of the manuscript.

## Supporting information



Supporting Information

## Data Availability

The data that support the findings of this study are available in the supplementary material of this article.
