# Supplementary material for: Chromium Cation‐Induced Self‐Reconstruction of a Stable and High Performance Boride‐derived Electrocatalyst for Oxygen Evolution Reaction
Source: Small. 2025 Sep 24;21(45):e07475. doi: 10.1002/smll.202507475 (PMC12614133; doi:10.1002/smll.202507475)
Supplement: Supplementary file 1 — Supporting Information [file SMLL-21-e07475-s001.docx]

Supporting Information

**Chromium Cation-induced Self-reconstruction of a Stable and High Performance Boride-derived Electrocatalyst for Oxygen Evolution Reaction**

*Charles Otieno Ogolla^#^,^[1]*^ Max Kasper,^[1]^ Muhammed Fasil Puthiyaparambath,^[2]^ Nastaran Farahbakhsh,^[3]^ Ranjit Thapa,^[2]^* Maiyalagan Thandavarayan,^[4]^ Manuela S. Killian,^[3]^ Benjamin Butz,^[1]^ Jean Marie Vianney Nsanzimana^#[1],[3],[5]*^*

C. O. Ogolla, M. Kasper, N. Dr. M. F. Muhammed, Farahbakhsh, Prof. R. Thapa, Prof. T. Maiyalagan, Prof. M. S. Killian, Prof. B. Butz, Dr. J. M. V. Nsanzimana

[1] C. O. Ogolla, M. Kasper, Prof. B. Butz, Dr. J. M. V. Nsanzimana
Micro- and Nanoanalytics Group, University of Siegen
Paul-Bonatz-Straße 9-11, 57076 Siegen, Germany
**E-mail:** *charles.ogolla@uni-siegen.de, jean.nsanzimana@uni-siegen.de*

[2] Dr. M. F. Muhammed, Prof. R. Thapa
Department of Physics, & Centre for Computational and Integrative Sciences, SRM University AP
Amaravati 522240, Andhra Pradesh, India
**E-mail:** *ranjit.t@srmap.edu.in*

[3] N. Farahbakhsh, Prof. M. S. Killian, Dr. J. M. V. Nsanzimana
Chemistry and Structures of Novel Materials, University of Siegen
Paul-Bonatz-Straße 9-11, 57076 Siegen, Germany

[4] Prof. T. Maiyalagan
 Department of Chemistry, SRM Institute of Science and Technology,

Kattankulathur, Tamilnadu, 603203 India

[5] Dr. J. M. V. Nsanzimana
Department of Industrial Engineering, University of Padova
Via Marzolo 9, 35131 Padua (PD), Italy

**[#] These authors contributed equally to this work.**

**Experimental *Section***

### **Materials**

The deionized water with a MilliQ filtration unit (conductivity σ = 18.2 M Ω∙cm) was used in this work. The other chemicals, such as sodium borohydride (NaBH_4_, Carl Roth, ≥97%), cobalt (II) chloride hexahydrate (CoCl_2_ . 6H_2_O, ChemSolute, ≥99%), sodium hydroxide (NaOH, Carl Roth, ≥99%), iron (III) chloride hexahydrate (FeCl_3_ . 6H_2_O, Merck, ≥99%), Chromium (III) chloride hexahydrate (CrCl_3_ . 6H_2_O, Thermo Scientific, ≥98%), manganese (II) chloride tetrahydrate (MnCl_2_ . 4H_2_O, Merck ≥99%), nickel (II) chloride hexahydrate (NiCl_2_ . 6H_2_O, Merck, ≥98%), zinc chloride (ZnCl_2_, Merck, ≥99%), hydrazine solution 35 wt% in water (N_2_H_4_, Sigma Aldrich), hydrogen peroxide 30% in water (H_2_O_2_, Merck), graphene platelets (iolitec, 6-8 nm), sulphuric acid (H_2_SO_4_, Merck, 95-97%) and hexamethylentetramin (C_6_H_12_N_4_, Merck, ≥99%) were used as received. Toray Carbon paper and Ni foil (purity: 99.99+ %, metal base) were purchased from Thermo Scientific and HMW Hauner GmbH & Co.KG, respectively, and they were cleaned before usage. The solvent ethanol (EtOH, VWR chemicals, ≥99,5% denaturated with 1% methyl ethyl ketone) was also used as-received.

**Synthesis of materials**

***Synthesis of binary metal borides:*** The metal borides were prepared by chemical reduction of transition metal salt in aqueous solution by sodium borohydride.^[1]^ To prepare Nickel boride, 10 mmol of the desired metal salts NiCl_2_ . 6H_2_O, Merck, ≥98% was dissolved in 50 mL of Milli-Q water. In a separate beaker, a sodium borohydride (NaBH_4_, 20 mmol, 756.6 mg) was dissolved in the NaOH solution (0.1 M, 20 mL) purged with N_2_, and the obtained solution was then transferred to a syringe and added drop by drop to the metal salt solution while stirring in an ice-water bath. After adding the NaBH_4_ solution, the reaction mixture was stirred for an additional 30 minutes to complete the reaction.

For binary boron-reduced iron and chromium samples, ethanol was used as a medium for dissolving the CrCl_3_ . 6H_2_O and FeCl_3_ . 6H_2_O, respectively. This followed a similar method described for the synthesis of Ni_2_B. For purification, the particles were collected in a centrifuge vial and washed 3 times with water and 3 times in ethanol before drying overnight to obtain powder catalysts for characterization.

***Synthesis of ternary to quaternary metal borides*:** For the synthesis of ternary and quaternary metal borides, a total of 10 mmol of the metal salts with the desired composition (FeNiB, CrNiB, CoNiB, MnNiB, and ZnNiB (χ_M_ = 0.2, where M is Fe, Cr, Co, Mn, Zn) or three-metal sources (Cr_y_Fe_z_Ni_x_B, where x, y, and z are the ratio of moles for metal salts in the solution) for ternary and quaternary borides, respectively) were dissolved in the water. In a separate beaker, a sodium borohydride (NaBH_4_, 20 mmol, 756.6 mg) was dissolved in the NaOH solution and this solution was then transferred to a syringe and added drop by drop to the metal salt solution while stirring in an ice-water bath. The remaining experimental procedures were repeated as reported in the section on the synthesis of the binary metal borides.

***Synthesis of Cr_0.15_Fe_0.15_Ni_0.7_-Oxide (CrFeNiO_x_) Nanocomposites:*** The nickel-iron-chromium oxide was synthesized by using a modified procedure reported by Siva et. al during the synthesis of nanostructured CuO/ZnO composite.^[1]^ In a dry beaker, 7 mmol of NiCl_2_ . 6H_2_O, along with 1.5 mmol of FeCl_3_ . 6H_2_O and 1.5 mmol of CrCl_3_ . 6H_2_O, were initially dissolved in 20 mL of water and stirred for 30 minutes. Subsequently, 10 mmol of NaOH (400 mg) were dissolved in 10 mL of water and added to the metal salt solution. The resulting solution was then transferred to an autoclave, sealed, and maintained at 180°C for 6 hours. For purification, the precipitates were transferred to a centrifuge tube. The particles were separated by centrifugation, removing the excess solution, and subsequently redispersed in water. This step was repeated three times using water and two times with ethanol. Finally, the particles were subjected to calcination at 400°C for 5 hours.

***Synthesis of Cr_0.15_Fe_0.15_Ni_0.7_ alloy (CrFeNi alloy):*** The nickel-iron-chromium alloy was synthesized by using a modified procedure reported during the synthesis of iron-nickel-cobalt alloy.^[2]^ Before the reaction, 50 mL of Milli-Q water was purged with argon in a 2-necked round-bottom flask equipped with a magnetic stirrer for 30 minutes. Afterward, 7 mmol of nickel chloride (NiCl_2_ . 6 H_2_O), each 1.5 mmoles of chromium chloride (CrCl_3_ . 6H_2_O) and iron chloride (FeCl_3_ . 6H_2_O), were added to this flask. In a separate beaker, 1812 mL of a 35 wt% hydrazine solution (20 mmol, 2 mol equivalents) was added and filled up to 20 mL. To this solution, 80 mg of sodium hydroxide (NaOH, 2 mmol) was added, and then this solution was purged for 30 minutes with nitrogen. The hydrazine solution was added to the metal salt solution by a syringe, and immediately a brownish precipitate formed in a strong exothermic reaction. The solution was maintained to 80 °C for half an hour, and afterward, the precipitate was collected by centrifugation and washed 3 times with water before they were dried in a vacuum oven overnight at room temperature.

**Materials Characterization**

***Inductively coupled plasma atomic emission spectroscopy (ICP-OES):*** The Spectro Acros II ICP-OES instrument from SPECTRO Analytical Instruments GmbH, (Kleve, Germany) was used throughout this study. The Nebulization was done with a micro-mist cross-flow nebulizer with a cyclonic spray chamber, and a fixed torch (30 mm) also from SPECTRO Analytical Instruments GmbH, (Kleve, Germany) was used. Argon gas (99.99% purity) obtained from Messer Industriegase (Siegen, Germany) was used as plasma gas. The Smart Analyzer Vision from the SPECTRO Analytical Instruments GmbH was used for controlling analysis. The axial configuration was employed to obtain maximum emission intensities.

***X-ray photoelectron spectroscopy (XPS):*** The samples were examined with a S-Probe ESCA SSX-100 S-probe photoelectron/ESCA spectrometer, utilizing Al Kα radiation. Analysis of the spectra was carried out using Casa XPS processing software version 2.3.16 PR 1.6. The aliphatic C 1s signal (284.8 eV) served as a reference for calibrating the binding energies. Before XPS data collection, the Ni foil was first rinsed with ethanol and deionized water in an ultrasonic bath for 10 min each. Inside the XPS vacuum chamber, the surface was further cleaned to remove the native NiO layer using Ar⁺ ion sputtering at 5 kV and 4 mA for 5 min under ultra-high vacuum conditions (around 10^-8^mbar). The high-resolution Ni 2p spectra were obtained immediately after sputtering to prevent re-oxidation.

***Powder X-ray diffraction (XRD):*** XRD analysis was performed using Panalytical x′pert pro diffractometer with a Cu K_α_ source. The sample to be analyzed was placed in a sample holder associated with the device and subsequently elevated by compression to avoid height discrepancies.

***Transmission electron microscopy (TEM):*** TEM analyses: (bright-field imaging (BFTEM), selected area electron diffraction (SAED) imaging, scanning transmission electron microscopy (STEM) imaging, and spectroscopic analysis (STEM-EELS), were done on a Thermo Fisher (FEI) Talos F200X. This instrument has a high-brightness XFEG gun and was run at an acceleration voltage of 200 kV. The instrument has a post column Gatan Continuum ER spectrometer which has a high speed DualEELS and DigiScan. The TEM is fitted with a generation 2 Super-X high solid angle EDXS detector.

***Scanning TEM - Electron energy loss spectroscopy (STEM – EELS):*** The STEM-EELS data was obtained by configuring the GIF spectrometer to the spectrum image acquisition mode, enabling DualEELS collection. EELS mappings were performed on representative areas of the samples. Dual EELS mappings, with a step size of approximately 2 nm, were recorded to capture core loss spectra within the energy loss range of 200–1600 eV (dispersion 0.75 eV/channel, convergence angle of 10.5 mrad and an acceptance angle of 42.01 mrad). This allowed for the identification of key ionization edges of B, C, O, Cr, Fe, and Ni at around 188, 285, 532, 575, 708, and 855 eV respectively. The low loss energy regime offset was set to 0 eV to correct the zero loss peak and address multiple scattering within the core-loss spectral regime. Constituent elements were quantified and mapped using theoretical cross-sections as implemented within the Gatan Digital Micrograph software suite (DM) version 3.60. Background correction was carried out using a power law function, and multiple scattering was removed through deconvolution using the Fourier-ratio method implemented in DM.

**Electrochemical evaluation**

***Preparation of working electrode:*** The catalyst was loaded on the working electrode, by dispersing 10 mg of catalyst in a mixture containing 950 μL EtOH, 20 μL H_2_O, and 30 μL Nafion solution, followed by 5 minutes of sonification in an ultrasonic bath to prepare a homogeneous ink. A disc-shaped glassy carbon electrode as RDE of a geometric area of 0.196 cm^2^ was used. Here, the loading of the catalyst was 0.3 mg cm^-2^. The resulting film was left to dry in air under ambient conditions for at least 20 min. In some measurements, 200 μL of the suspension was drop cast on each side of the carbon paper substrate (1.0 cm^-2^), to yield a final concentration of 2 mg cm^-2^.

***Electrochemical measurements:*** All the electrochemical measurements were carried out using a potentiostat (Biologic SP-200) in a three-electrode configuration, equipped with a built-in EIS analyzer. A rectangular Toray Carbon paper of geometric area 1 cm^2^ (thickness 0.2 mm) loaded with the corresponding catalyst was used as the working electrode, a graphite cylindrical electrode as the counter electrode, and a Hg/HgO as the reference electrode. An electrolyte solution with a concentration of 1 M KOH.

Before the OER measurements, the electrodes were subjected to continuous potential cycling in the potential from 0.9 V to 1.8 V vs RHE with a scan rate of 100 mV s^-1^ until reproducible voltammograms were obtained. All measured potentials were converted to the reversible hydrogen electrode (RHE), according to the Nernst Equation:

| $E_{RHE}=E_{Hg/HgO}+0.0983+0.0592*pH$ | (1) |
| --- | --- |

All obtained polarization curves were corrected for ohmic losses (including the wiring, solution, substrate, and catalyst resistance). The correction was performed according to the equation E = E_RHE_ - iR_s_. The resistivity $R_{\mathrm{solution}}$ (R_s_) and charge transfer resistance $R_{\mathrm{ct}}$ was obtained from an EIS Nyquist plot.

The EIS measurement was performed at a potential of 0.6 V in the frequency range of 100 kHz to 1 Hz. The LSV curves were recorded with a scanning rate of 5 mV s^-1^ and the Tafel slope was obtained by linear fitting the overpotential against the base-10 logarithm of the current density. To depict the Tafel slope value, the following Tafel equation: *η* = *a* + *b* log *j*, where b is the Tafel slope and j is the current density.

***Electrochemical Impedance Spectroscopy (EIS) measurement:*** The ECSA was determined by measuring the chemical double-layer capacitances $C_{\mathrm{dl}}$ from the scan rate CV-dependence plot. CV scans were performed at different scan rates (40, 60, 80, 120, 160 & 200 mV sec^-1^) in the non-Faradaic potential region (usually 0.05 V to 0.15 V vs RHE) and $\Delta j=\frac{1}{2}\left( j_{\mathrm{charge}}-j_{off charge} \right)$ was estimated at the average potential in the selected range. The slope obtained by linear fitting of $\Delta j$ against the different scan rates yields the double-layer capacitance $C_{\mathrm{dl}}$ value. The ECSA was calculated using equation 2. A specific capacitance (C_s_) of 40 μF cm^-2^.

ESCA = C_dl_/C_s_ (2)

**Turnover frequency (TOF):** The TOF is defined as the rate of oxygen molecules produced per active site and can relate to the intrinsic activity of the electrodes. It can be calculated by the following equation.^[3]^

| $TOF=\frac{I}{4nF}$ | (3) |
| --- | --- |

Here $n$ represent the number of moles of the active sites, the term 4 corresponds to the number of electrons involved in the OER and $F$ indicates the Faraday constant (96,485 C mol^−1^). The determination of $n$ was performed using the following formula:^[3]^

| $n=\frac{Q}{F A}$ | (4) |
| --- | --- |

Here $A$ represent the testing are of the electrode and $Q$ denotes the total charge of the redox peaks, which can be determined by integrating the first forward sweeps of a cyclic voltammetry (CV) scan in the active region.^[3]^ The CV scan was performed in the range of 0.5 V to 0.655 V with a scan rate of 100 mV s^-1^. The recyclability and stability behavior of the catalyst was established by conducting 1000 CV cycles and applying 10 mV cm^-2^ for 24 h, respectively.

**Theoretical evaluations**

Density Functional Theory (DFT) calculations were carried out using the Vienna *Ab-initio* Simulation Package (VASP),^[4, 5]^ to investigate the underlying reason for the enhanced activity upon Cr incorporation into the metal boride complex. The crystal structure of Ni_2_B was optimized, giving lattice parameters of *a* = *b* = 4.93 Å and *c* = 4.21 Å, which are consistent with previously reported values.^[6]^ To model the B-O and metal-O interactions consistent with the experimental observations, the O atom was preferentially adsorbed on the surface boron termination. The Fe_0.40_Ni_1.6_B composition was constructed by substituting five Ni atoms with Fe in a Ni_24_B_48_ supercell, while the Cr_0.09_Fe_0.41_Ni_1.5_B composition was obtained by replacing one of the Ni atoms with a Cr atom in Fe_0.40_Ni_1.6_B.

The free energy $(\Delta G$) and overpotential for the OER were calculated using the 4 *e*^-^ mechanism proposed by Nørskov *et al*^[7]^ and is given by

$\Delta G = \Delta E + \Delta ZPE - T\Delta S + \Delta G_{pH}+ \Delta G_{U}$ (1)

Where $\Delta E$ is the total energy difference, $\Delta ZPE$is the zero-point energy, $T\Delta S$ is the entropy correction term, $\Delta G_{pH}$ is the effect of pH and $\Delta G_{U}$ is the bias voltage.


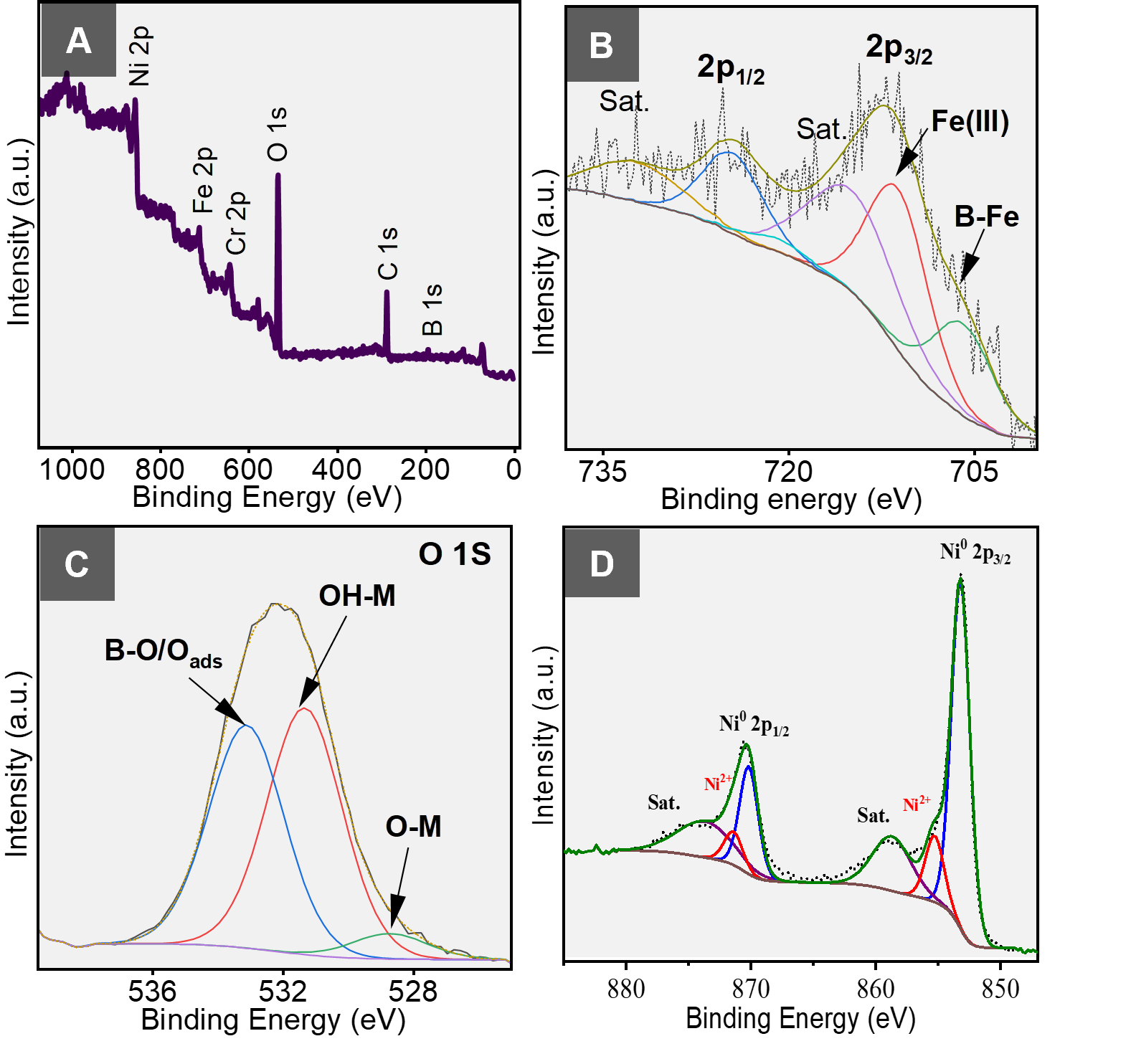


**Figure S1**: A) XPS survey. B-C) XPS spectra for Fe 2p and O 1s of Cr-FeNiB nanoparticles. D) XPS Ni 2p spectrum of clean Ni foil.


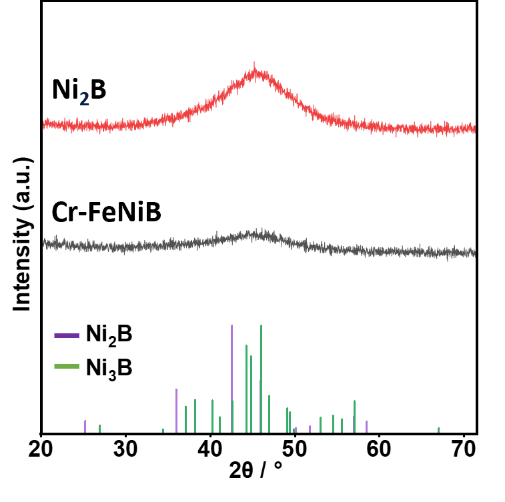


**Figure S2**: XRD pattern of Ni_2_B and Cr-FeNiB powders.

**
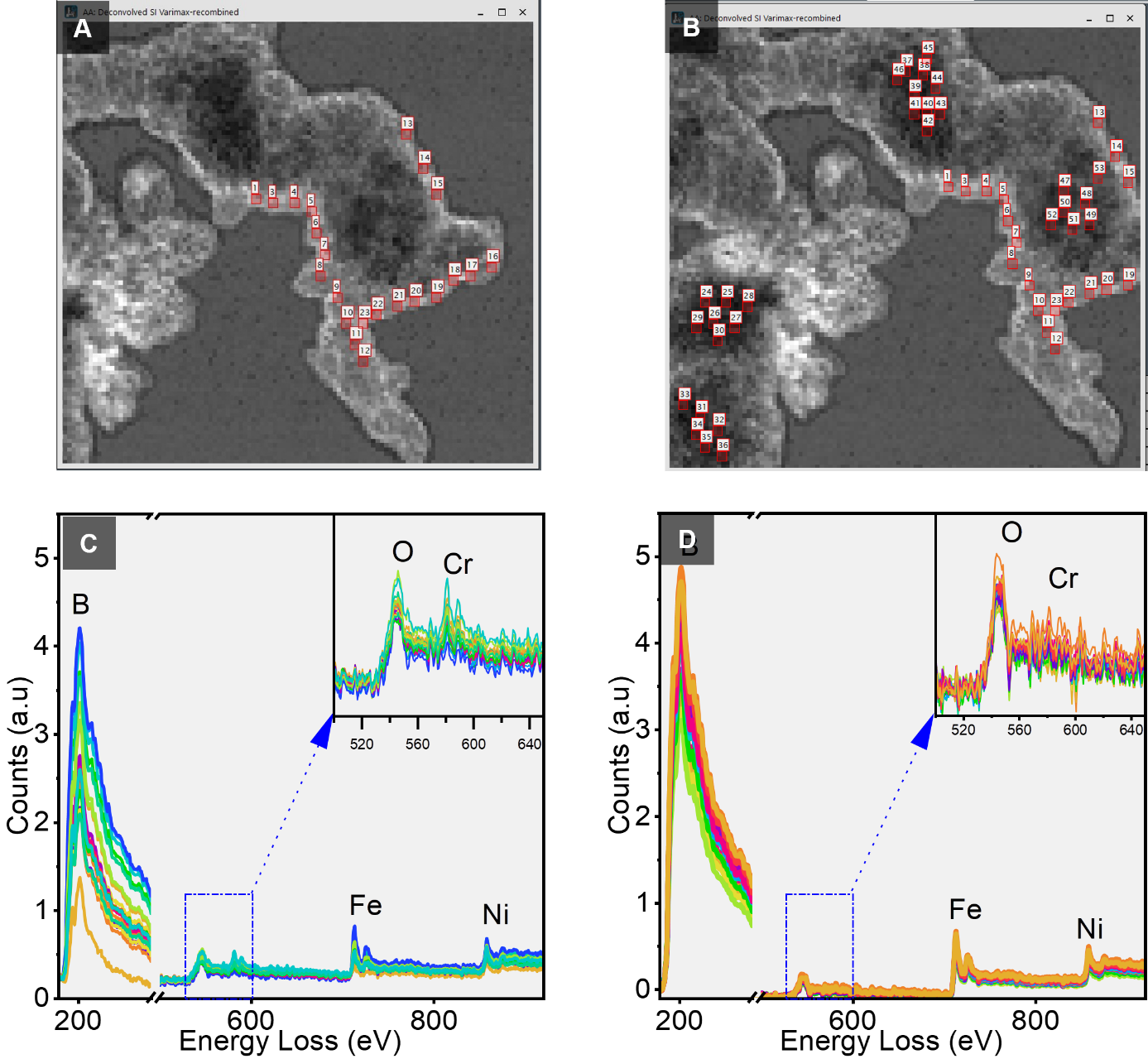
**

**Figure S3**: **Edge vs. bulk EELS composition analyses of as-prepared catalyst:** A & C) ROI selected from the rim region of the sample with overlaid spectra in C showing statistical agreement; B & D) ROI and spectra respectively of bulk from pre-catalyst.

**
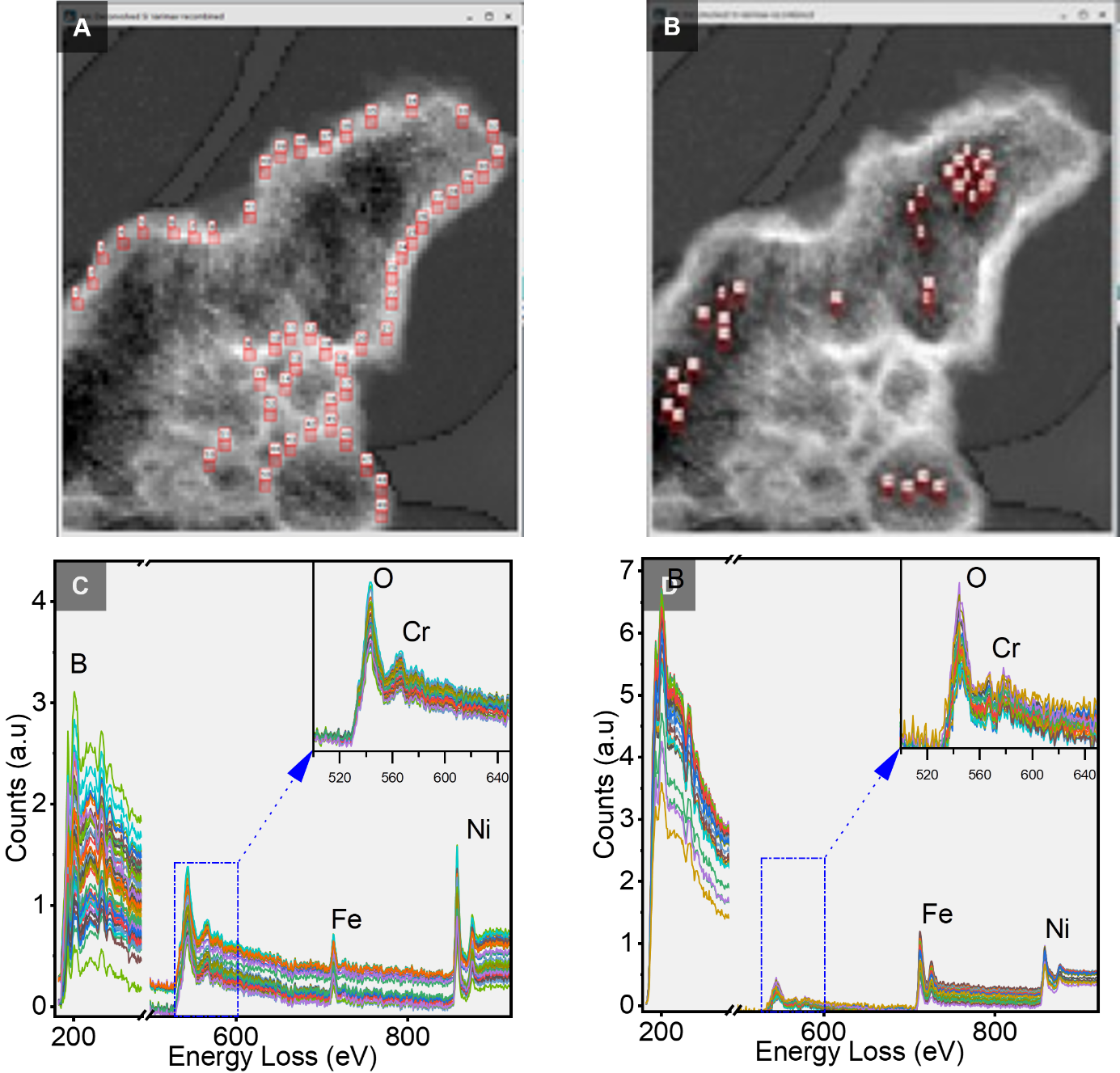
**

**Figure S4: Edge vs. bulk EELS composition analyses of catalyst:** A & C) ROI selected from the rim region of the sample with overlaid spectra in C showing statistical agreement; B & D) ROI and spectra, respectively, of bulk from catalyst.


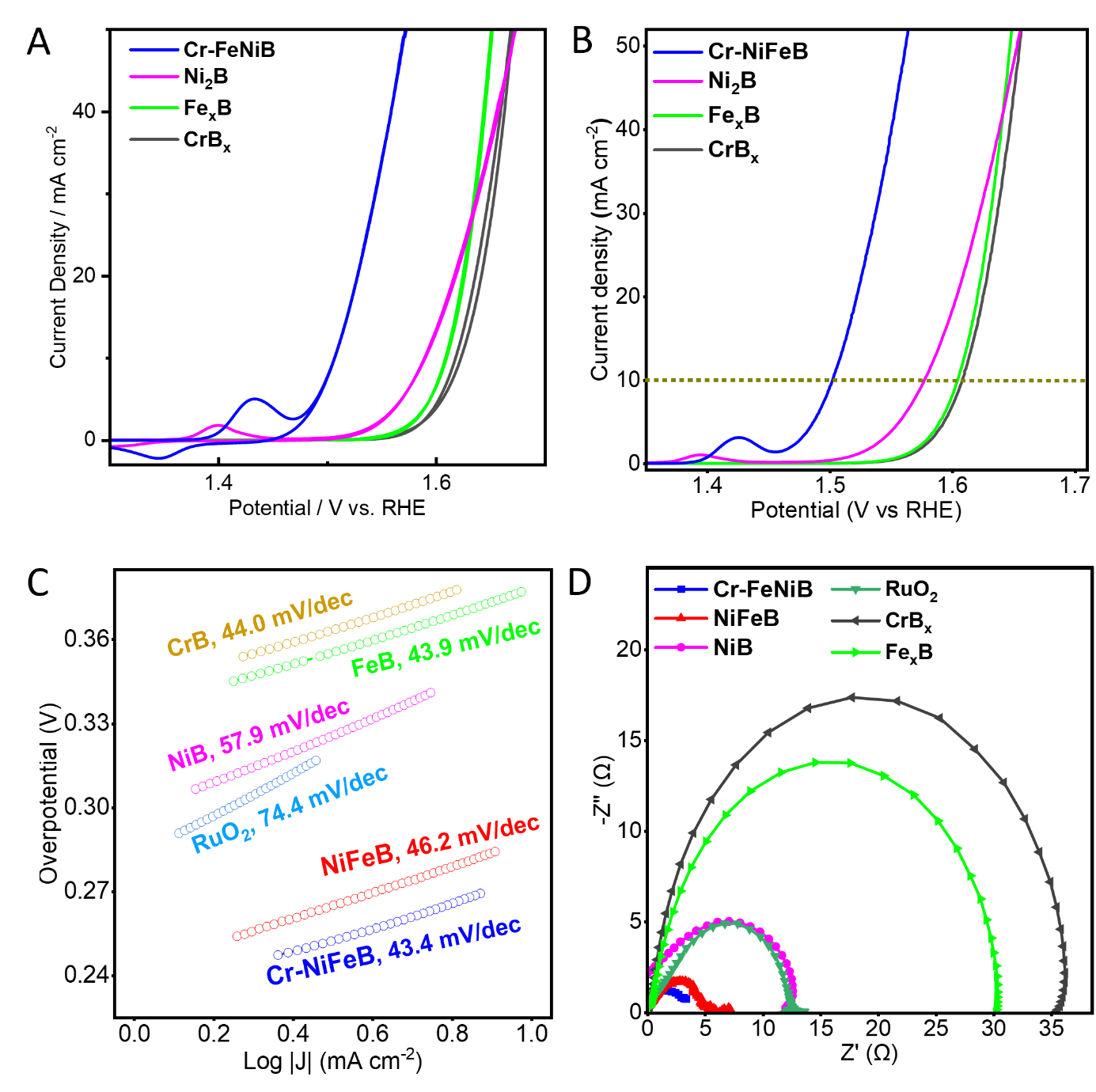


**Figure S5:** CV, LSV, Tafel slopes, and EIS for Cr-FeNiB, FeNiB, commercial RuO_2,_ and binary catalysts on glassy carbon.


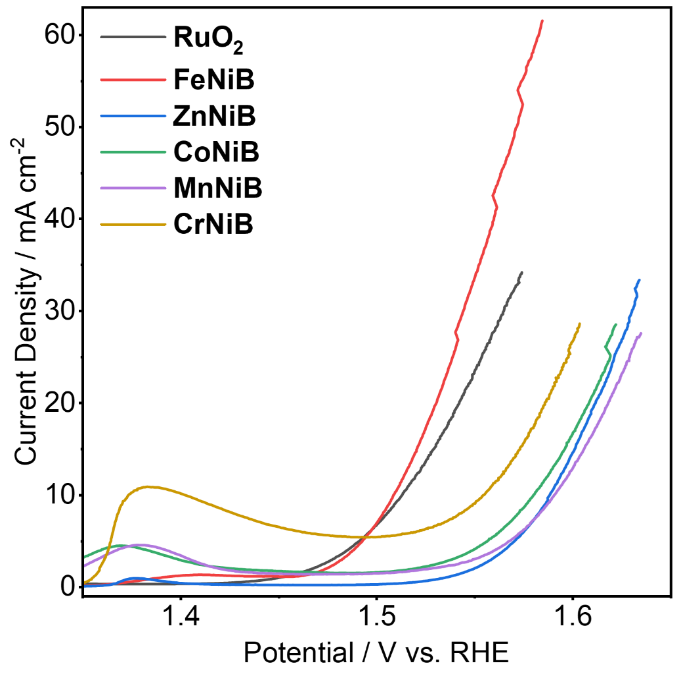


**Figure S6:** LSV for comparing ternary Ni-boride derived catalysts (FeNiB, CrNiB, CoNiB, MnNiB, and ZnNiB (χ_M_ = 0.2, where M is Fe, Cr, Co, Mn, Zn)) on carbon paper.


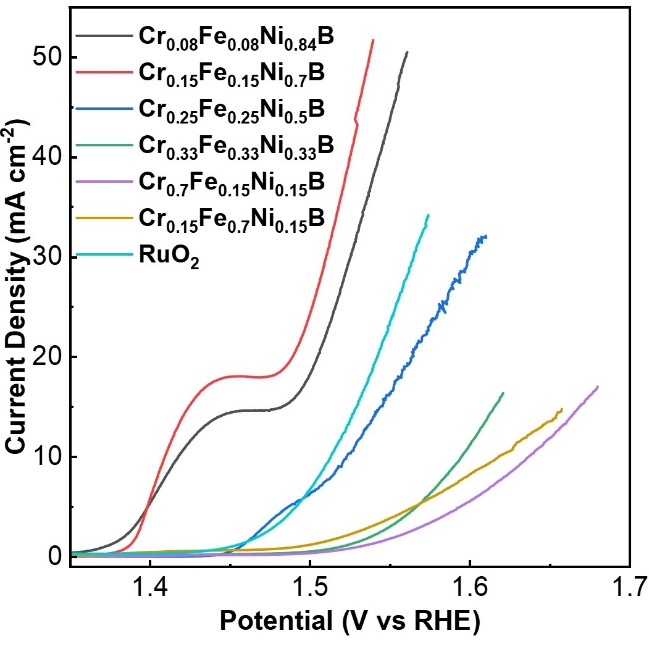


**Figure S7:** LSV for the optimization of quaternary Ni-boride derived catalyst on carbon paper. Ni_0.15_Cr_0.15_Fe_0.7_B and Ni_0.15_Cr_0.7_Fe_0.15_B were synthesized in EtOH, because otherwise hydroxide formation was observed.


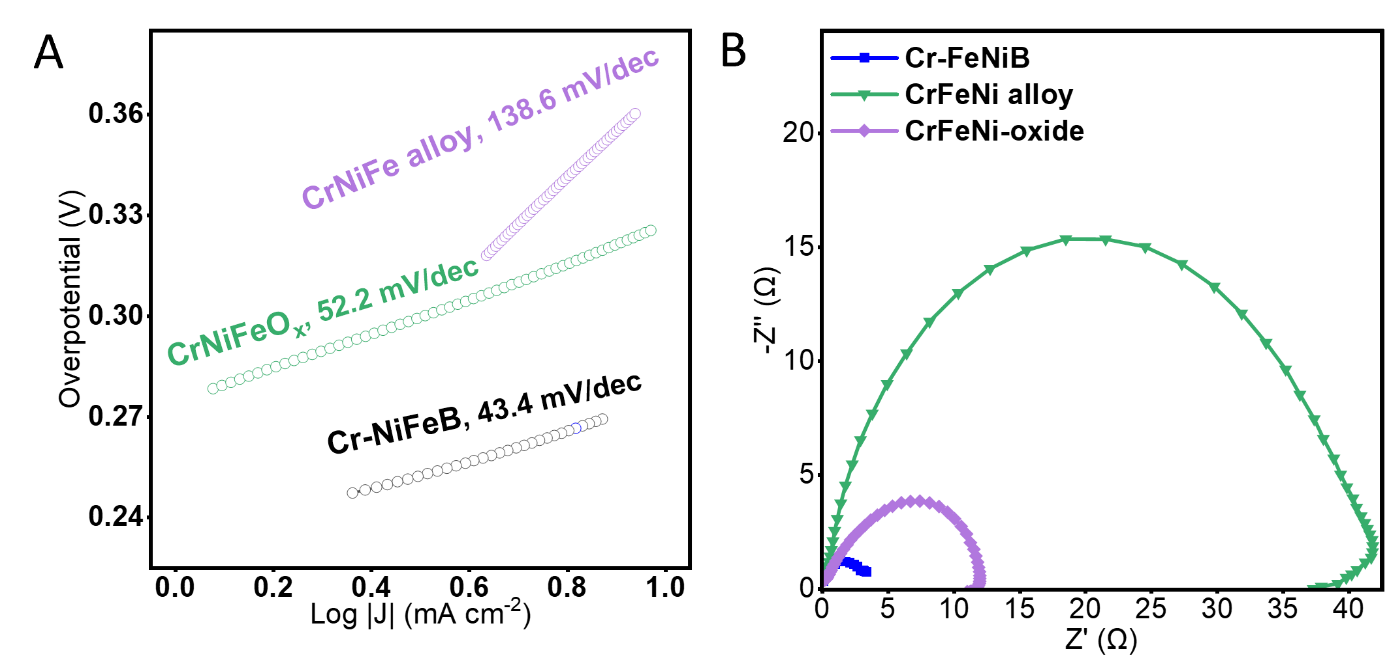


**Figure S8:** Tafel slopes and EIS analysis for Cr-FeNi boride, oxide, and alloy (metal salt ratio in precursor solution 1.5:1.5:0.7 for Cr, Fe, and Ni, respectively).


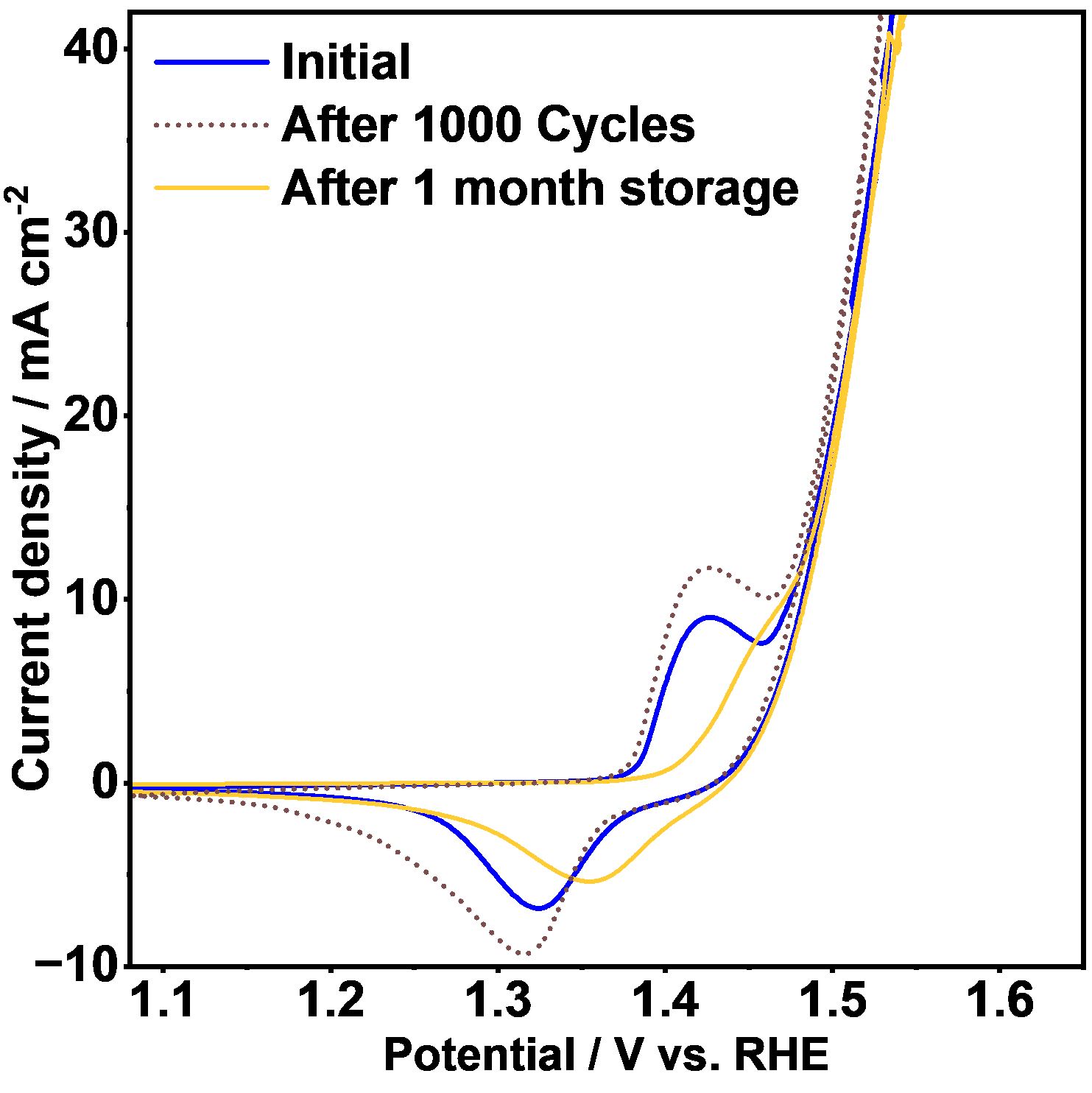


**Figure S9:** Initial CV and CV scans after 1000 CV cycles, as well as the CV scan of the sample after storage of the catalyst for one month at room temperature conditions and in air. The Cr-FeNiB achieved a current density of 10 mA cm^-2^ at 252 mV on carbon paper. This overpotential was retrieved from the backward scan of the CV.


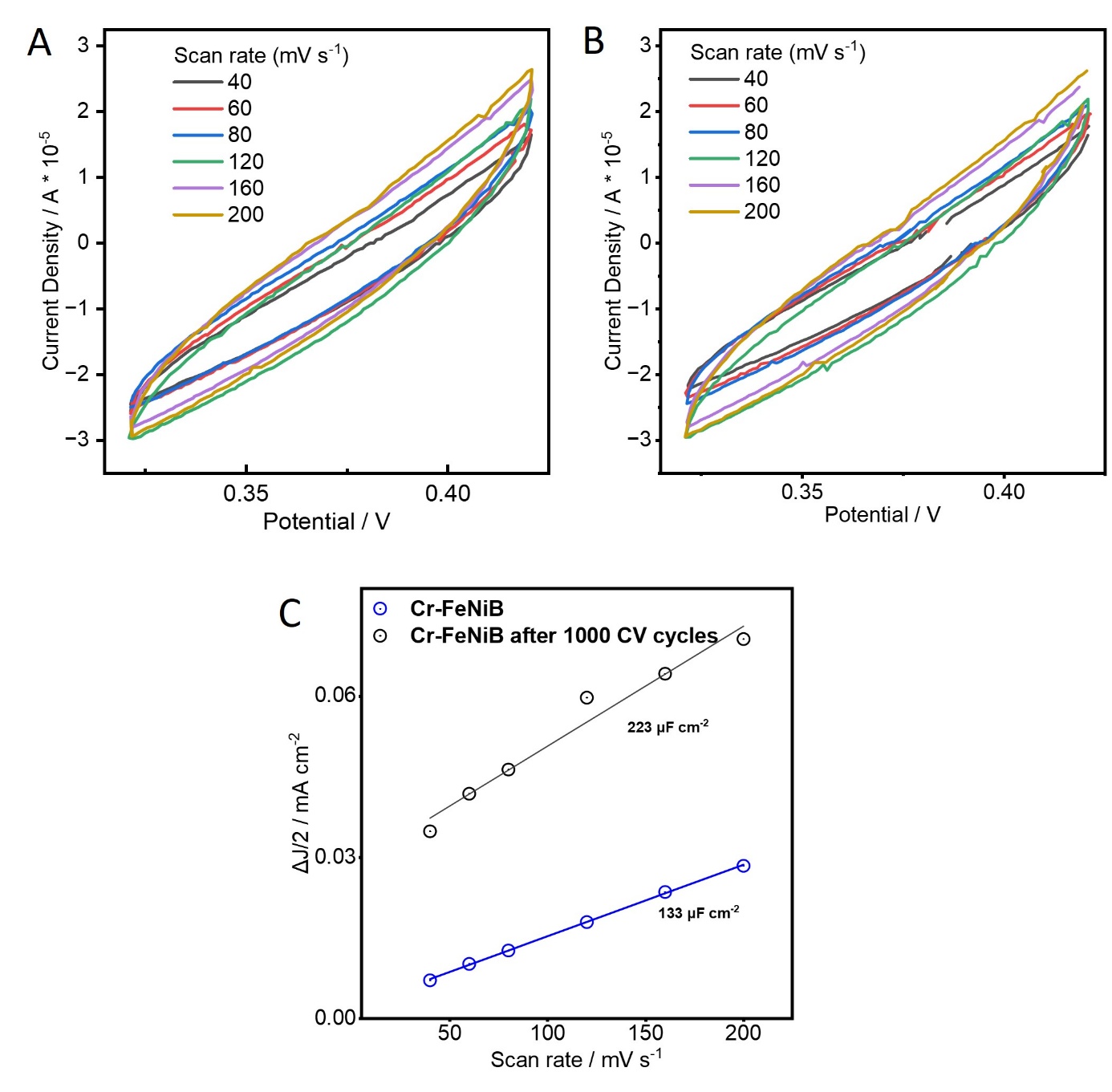


**Figure S10:** Estimation of C*_dl_* and calculation of ECSA of Cr-FeNiB prior (3.3) and after the 1000 CV cycles (5.5).


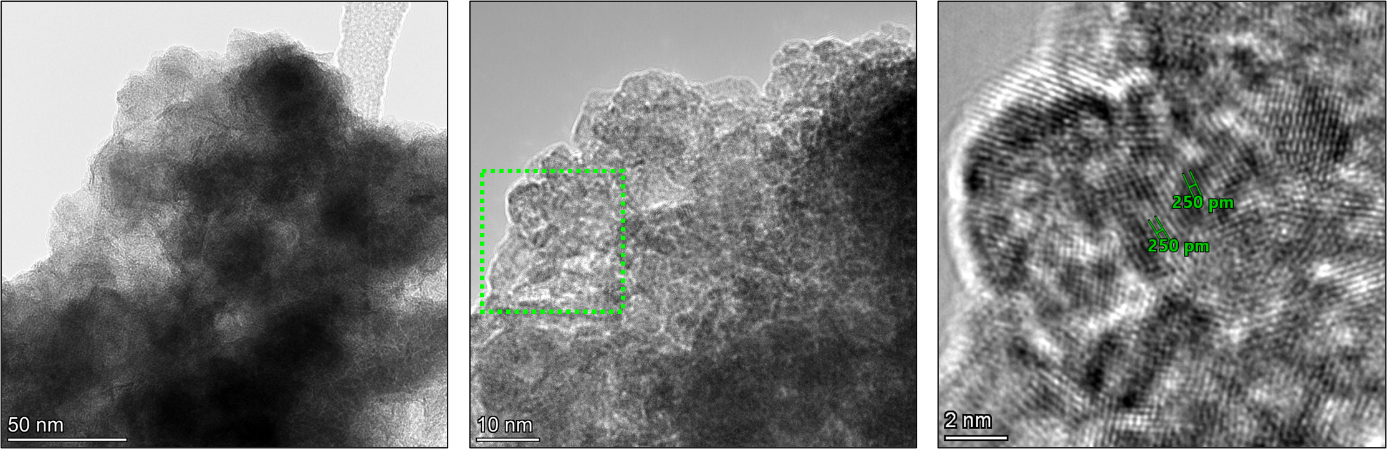


**Figure S11:** High resolution TEM images of Cr–FeNiB_1000CV_ showing exemplary lattice fringes within the nanocrystalline domains


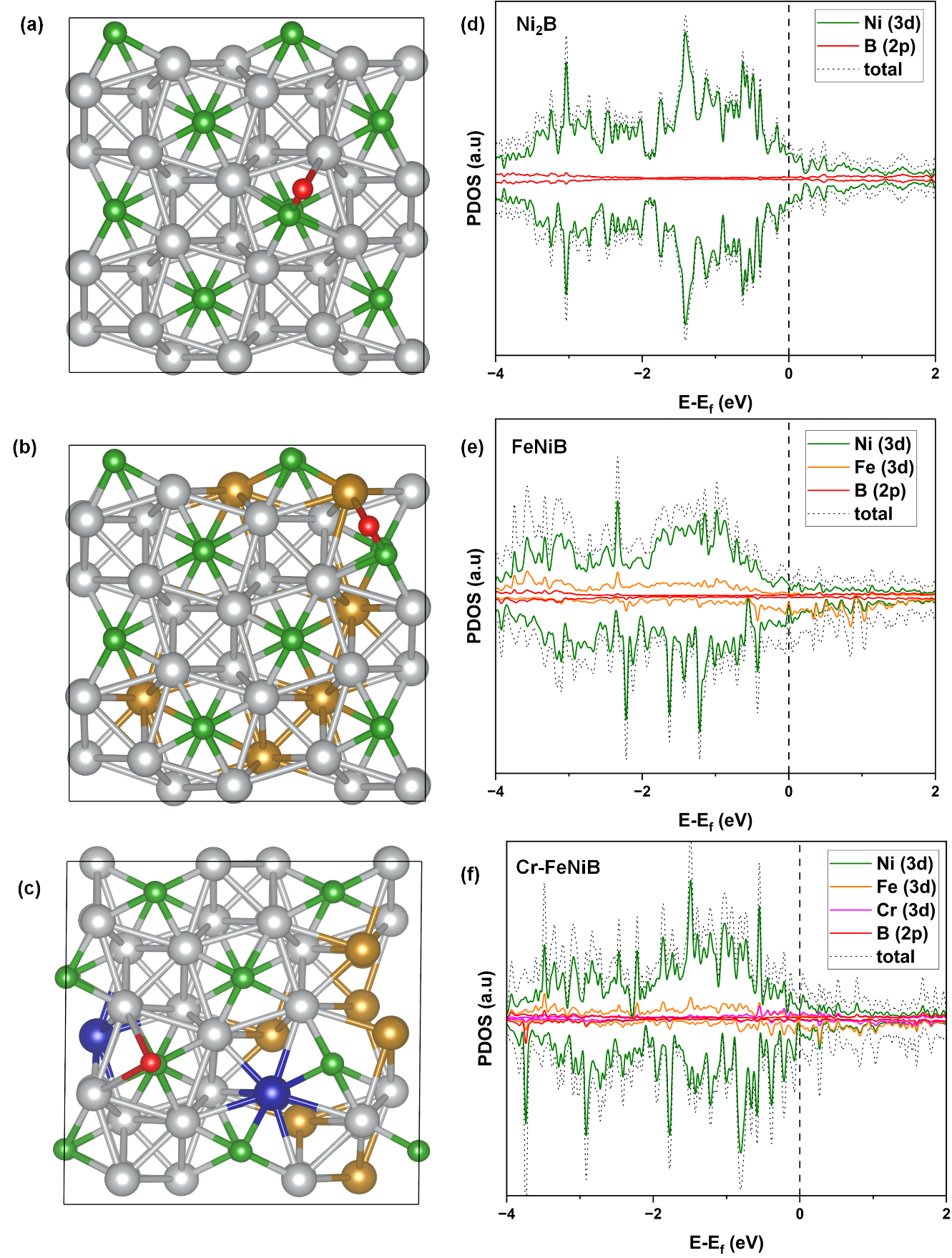


**Figure S12:** Optimized configuration and PDOS of (a) and (d) Ni_2_B, (b) and (e) FeNiB, (c) and (f) Cr-FeNiB, where Ni (grey), Fe (golden), Cr (violet), B (green) and O (red) atoms.

**References**

1. V. Siva, A. Murugan, A. S. Shameem, S. A. Bahadur, One-step hydrothermal synthesis of transition metal oxide electrode material for energy storage applications, *Journal of Materials Science: Materials in Electronics*, 2020, **31**, 20472-20484. <Https://doi.org/10.1007/s10854-020-04566-2>

2. Y. Yang, C. Xu, Y. Xia, T. Wang,F. Li, Synthesis and microwave absorption properties of FeCo nanoplates, *Journal of Alloys and Compounds*, 2010, **493**, 549-552. <https://doi.org/10.1016/j.jallcom.2009.12.153>

3. L. Bruno, M. Scuderi, F. Priolo,S. Mirabella, Enhanced electrocatalytic activity of low-cost NiO microflowers on graphene paper for the oxygen evolution reaction, *Sustainable Energy & Fuels*, 2022, **6**, 4498-4505. <Https://doi.org/10.1039/D2SE00829G>

4. G. Kresse,J. Furthmüller, Efficient iterative schemes for ab initio total-energy calculations using a plane-wave basis set, *Phys. Rev. B*, 1996, **54**, 11169-11186. <https://doi.org/10.1103/PhysRevB.54.11169>

5. G. Kresse,J. Furthmüller, Efficiency of ab-initio total energy calculations for metals and semiconductors using a plane-wave basis set, *Comput. Mater. Sci.*, 1996, **6**, 15-50. <https://doi.org/10.1016/0927-0256(96)00008-0>

6. C. T. Zhou, J. D. Xing, B. Xiao, J. Feng, X. J. Xie,Y. H. Chen, First principles study on the structural properties and electronic structure of X2B (X=Cr, Mn, Fe, Co, Ni, Mo and W) compounds, *Comput. Mater. Sci.*, 2009, **44**, 1056-1064. <https://doi.org/10.1016/j.commatsci.2008.07.035>

7. J. K. Nørskov, J. Rossmeisl, A. Logadottir, L. Lindqvist, J. R. Kitchin, T. Bligaard,H. Jónsson, Origin of the Overpotential for Oxygen Reduction at a Fuel-Cell Cathode, *J. Phys. Chem. B*, 2004, **108**, 17886-17892. <https://doi.org/10.1021/jp047349j>
